# High-Resolution CT Findings in Interstitial Lung Disease Associated with Connective Tissue Diseases: Differentiating Patterns for Clinical Practice—A Systematic Review with Meta-Analysis

**DOI:** 10.3390/jcm14176164

**Published:** 2025-08-31

**Authors:** Janet Camelia Drimus, Robert Cristian Duma, Daniel Trăilă, Corina Delia Mogoșan, Diana Luminița Manolescu, Ovidiu Fira-Mladinescu

**Affiliations:** 1 Doctoral School, “Victor Babes” University of Medicine and Pharmacy Timisoara, Eftimie Murgu Square 2, 300041 Timisoara, Romania; janet.drimus@umft.ro; 2 Center for Research and Innovation in Personalised Medicine of Respiratory Diseases (CRIPMRD), Pulmonology University Clinic, “Victor Babes” University of Medicine and Pharmacy Timisoara, Eftimie Murgu Square 2, 300041 Timisoara, Romania; mladinescu@umft.ro; 3 Department of Radiology & Medical Imaging, County Emergency Hospital “Pius Brinzeu” Timisoara, Liviu Rebreanu Boulevard 156, 300723 Timisoara, Romania; robertduma@yahoo.com; 4 2nd Pulmonology Ward, Center of Expertise for Rare Lung Diseases, Clinical Hospital of Infectious Diseases and Pneumophthisiology “Dr. Victor Babes” Timisoara, Gh. Adam Street No. 13, 300310 Timisoara, Romania; dmanolescu@umft.ro; 5 Rheumatology Department, “Carol Davila” University of Medicine and Farmacy, 020021 Bucharest, Romania; cmogosan@yahoo.com; 6 “Dr. Ion Stoia” Clinical Center for Rheumatic Diseases, 5th Thomas Masaryk Street, District 2, 020983 Bucharest, Romania; 7 Radiology and Medical Imaging, “Victor Babes” University of Medicine and Pharmacy Timisoara, Eftimie Murgu Square 2, 300041 Timisoara, Romania

**Keywords:** connective tissue diseases, interstitial lung disease, high-resolution computed tomography, imaging patterns, radiologic features, autoimmune lung involvement, rheumatologic lung disease, diffuse parenchymal lung disease

## Abstract

**Objectives**: Connective tissue diseases (CTDs) include a diverse group of systemic autoimmune conditions, among which interstitial lung disease (ILD) is acknowledged as a major determinant of prognosis. High-resolution computed tomography (HRCT) is the gold standard for ILD assessment. The distribution of HRCT patterns across CTDs remain incompletely defined. The objective of this systematic review is to synthesize available evidence regarding the prevalence of specific radiological patterns within CTD-ILDs and to assess whether specific patterns occur at different frequencies among individual CTDs. **Methods**: The inclusion criteria encompassed original human studies published in English between 2015 and 2024, involving adult participants (≥18 years) with CTD-ILDs assessed primarily by HRCT and designed as retrospective, prospective, or cross-sectional trials with extractable data. We systematically searched PubMed, Scopus, and Web of Science (January 2025). Risk of bias was evaluated using the Newcastle–Ottawa Scale (NOS) for cohort and case–control studies, and the JBI Critical Appraisal Checklist for cross-sectional studies. Data were extracted and categorized by HRCT pattern for each CTD, and then summarized descriptively and statistically. **Results**: We analyzed 23 studies published between 2015 and 2024, which included 2020 patients with CTD-ILDs. The analysis revealed non-specific interstitial pneumonia (NSIP) as the most prevalent pattern overall (36.5%), followed by definite usual interstitial pneumonia (UIP) (24.8%), organizing pneumonia (OP) (9.8%) and lymphoid interstitial pneumonia (LIP) (1.25%). HRCT distribution varied by CTD: NSIP predominated in systemic sclerosis, idiopathic inflammatory myopathies, and mixed connective tissue disease; UIP was most frequent in rheumatoid arthritis; LIP was more common in Sjögren’s syndrome. While global differences were statistically significant, pairwise comparisons often lacked significance, likely due to sample size constraints. **Discussion**: Limitations include varying risk of bias across study designs, heterogeneity in HRCT reporting, small sample sizes, and inconsistent follow-up, which may reduce precision and generalizability. In addition to the quantitative synthesis, this review offers a detailed description of each radiologic pattern mentioned above, illustrated by representative examples to support the recognition in clinical settings. Furthermore, it includes a brief overview of the major CTDs associated with ILD, summarizing their epidemiological data, risk factors for ILD and clinical presentation and diagnostic recommendations. **Conclusions**: NSIP emerged as the most common HRCT pattern across CTD-ILDs, with UIP predominating in RA. Although inter-disease differences were observed, statistical significance was limited, likely reflecting sample size constraints. These findings emphasize the diagnostic and prognostic relevance of HRCT pattern recognition and highlight the need for larger, standardized studies.

## 1. Introduction

### 1.1. Background: Brief Overview of Connective Tissue Diseases (CTDs) and the Importance of High-Resolution Computed Tomography (HRCT)

Connective tissue diseases (CTDs) are a group of disorders characterized by immune-mediated multi-organ dysfunction and heterogeneous systemic features [1]. The pathophysiology of CTDs involves a dysregulated immune response, wherein a loss of self-tolerance leads to the production of autoantibodies and immune-mediated damage to structural components of connective tissues. This leads to inflammation and damage in various organs, including the skin, joints, and internal organs [1]. The main types of CTDs include Systemic Sclerosis (SSc), Rheumatoid Arthritis (RA), Idiopathic Inflammatory Myopathies (IIMs—such as polymyositis and dermatomyositis), Sjogren’s Syndrome (SS), Systemic Lupus Erythematosus (SLE), and Mixed Connective Tissue Disease (MCTD). Lung involvement, particularly interstitial lung disease (ILD), is a major contributor to mortality in diseases like SSc and is an increasing cause of death in RA [2].

High-resolution computed tomography, commonly abbreviated as HRCT, constitutes an indispensable and critical tool in the medical diagnostic process, particularly in the identification and assessment of ILD. By recognizing specific morphological patterns, HRCT helps differentiate between various CTDs and tailor appropriate treatment strategies [3,4]. A clear understanding of HRCT findings enables clinicians to evaluate the degree of lung involvement and fibrosis, which is important for assessing disease severity and tracking progression over time [4]. Since different HRCT patterns are associated with varying prognoses and treatment responses, accurate interpretation is needed to optimize patient care [5]. Additionally, it aids in distinguishing CTD-ILDs from other conditions, ensuring accurate diagnosis and management [6].

### 1.2. Objective of This Review

This paper aims to provide an in-depth insight into HRCT findings in CTDs, with a specific emphasis on distinguishing ILD patterns that are associated with them. Particular attention will be directed toward the identification of morphological patterns, including non-specific interstitial pneumonia (NSIP), usual interstitial pneumonia (UIP), organizing pneumonia (OP), and lymphoid interstitial pneumonia (LIP), because certain HRCT patterns tend to predominate in specific CTDs.

The objective of this systematic review is to synthesize available evidence regarding the prevalence of specific radiological patterns within CTD-ILDs and to assess whether specific patterns occur at different frequencies among individual CTDs.

## 2. Materials and Methods

### 2.1. Data Sources and Search Strategy

This review was conducted following the PRISMA (Preferred Reporting Items for Systematic Reviews and Meta-Analyses) guidelines [7]. We performed a comprehensive search of the literature using PubMed, Scopus and Web of Science databases in January 2025, which included key terms such as, but not limited to, the following: “Connective Tissue Disease”, “Rheumatoid Arthritis”, “Systemic Sclerosis”, “Sjögren’s Syndrome”, “Systemic Lupus Erythematosus”, “Polymyositis”, “Dermatomyositis”, “Mixed Connective Tissue Disease”, “Interstitial Lung Disease”, “Diffuse Parenchymal Lung Disease”, “HRCT”, “High-Resolution Computed Tomography”, “Radiological Features”, “Imaging Patterns”, “Ground-Glass Opacity”, “Honeycombing”, “Reticulation”, “Non-Specific Interstitial Pneumonia”, “NSIP”, “Usual Interstitial Pneumonia” and “UIP”. The search was adjusted as needed for each database.

### 2.2. Inclusion and Exclusion Criteria

The initial stage of the review process consisted of a single screening at the title and abstract level (see Table 1 for the detailed inclusion and exclusion criteria), followed by a more detailed assessment in which selected studies were included and further analyzed for eligibility.

### 2.3. Risk of Bias

Risk of bias for the included studies was evaluated using standardized tools according to study design. For cohort and case–control studies, we applied the Newcastle–Ottawa Scale (NOS), which assesses three domains: selection of participants, comparability of study groups, and ascertainment of exposure/outcome. For cross-sectional studies, we used the Joanna Briggs Institute (JBI) Critical Appraisal Checklist, which evaluates methodological quality across domains such as sampling, measurement of exposure and outcome, identification and management of confounding factors, and appropriateness of statistical analysis.

Risk of bias assessment was independently performed by two reviewers (J.D. and R.D.), with disagreements resolved by discussion or, if needed, were arbitrated by a third reviewer (D.T.). A complete summary of the risk of bias assessment for each study is provided in the Supplementary Material (Tables S1–S3).

We planned to assess both the risk of reporting bias and the certainty of the evidence for each CTD. Due to the limited number of studies per connective tissue disease and the heterogeneity of study designs and populations, formal statistical assessment of reporting bias (e.g., funnel plots, Egger’s test) and a structured GRADE evaluation were not feasible.

### 2.4. Data Collection

Data from the included studies were extracted using a standardized form developed for this review by the authors mentioned above. We extracted data on study characteristics (author, year of publication, country, study design, sample size) and HRCT findings. The main outcome of interest was the distribution of HRCT patterns across different connective tissue diseases (CTDs). Extracted information was categorized by HRCT pattern for each connective tissue disease (CTD). When non-UIP groups were reported but not otherwise specified, these were reclassified as “Other.” If multiple reports from the same cohort were identified, only the report providing the most complete and relevant HRCT data was included in the synthesis. When data for a particular HRCT pattern or CTD subgroup were not reported, only the available information was extracted. No additional data were obtained from study authors. The extracted data were then compiled in Microsoft Excel, and analyses and visualizations were performed using MedCalc (Version 23.1.3). To explore heterogeneity in the distribution of HRCT patterns across CTDs, we performed non-parametric group comparisons. The Kruskal–Wallis test was used to assess overall differences among CTD subgroups, followed by post hoc Dunn’s tests for pairwise comparisons with adjustment for multiple testing. No additional sensitivity or subgroup analyses were performed.

No conventional effect measures were used, as this review focused on descriptive imaging outcomes rather than intervention effects.

## 3. Results

### 3.1. Search Results

A total of 985 records were identified through the search strategy. All records were imported into Zotero (Version 7.0.24) reference management software, where automatic and manual duplicate removal was performed. After removal of 398 duplicate or clearly ineligible records, 587 records remained for screening.

Two reviewers (J.D. and R.D.) independently screened titles and abstracts of the 587 records against the predefined eligibility criteria. Any discrepancies were resolved by discussion. Where consensus could not be reached, a third reviewer (D.T.) adjudicated to make the final decision. Following this process, 256 records were excluded.

**Figure 1:**
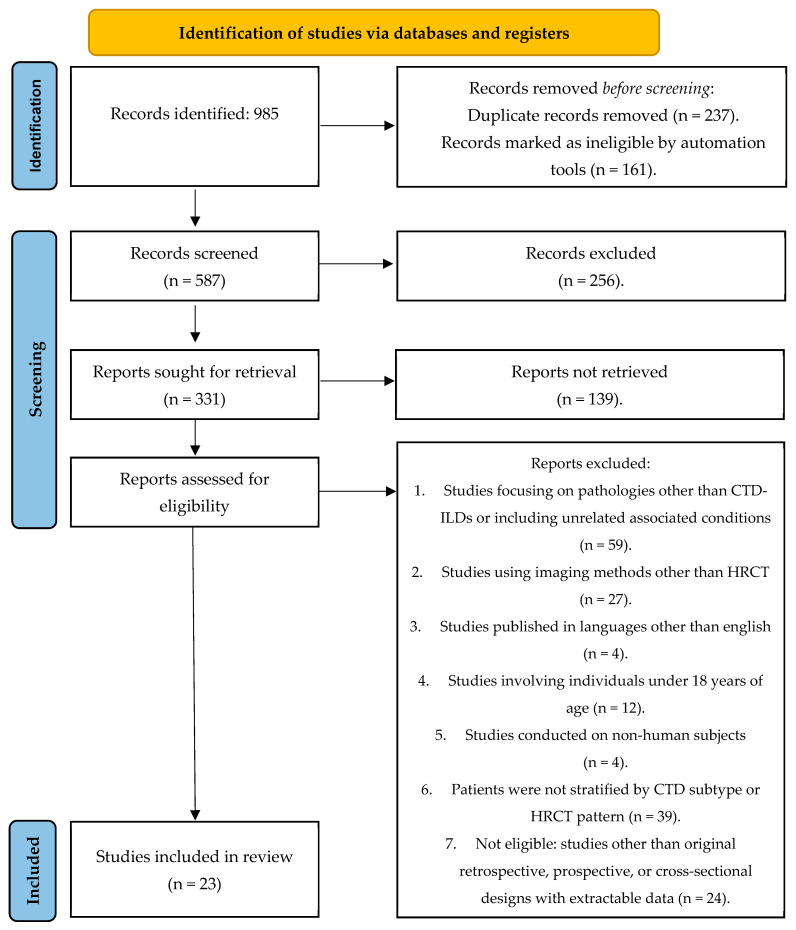
PRISMA 2020 flow diagram—literature review of studies published between 2015 and 2024 [7].

Full-text articles were sought for 192 potentially eligible studies; of these, 139 could not be retrieved. The remaining 53 full-text articles were independently assessed for eligibility by two reviewers (J.D. and R.D.). Disagreements were resolved by discussion, with involvement of the third reviewer (D.T.) if necessary. No studies were excluded after full-text assessment that appeared to meet the inclusion criteria. Ultimately, 23 studies met the inclusion criteria and were included in this review. The PRISMA flowchart below illustrates the search results and the step-by-step process of study exclusion (Figure 1).

### 3.2. Literature Review

We analyzed 23 studies published between 2015 and 2024, which included 2020 patients with CTD-ILDs (141 SSc, 1082 RA, 167 SS, 559 IIMs, 71 MCTD). These articles were selected and systematically assessed according to the HRCT patterns observed in each specific CTD: UIP, probable UIP, indeterminate for UIP, NSIP, OP, LIP and others (which included combinations of different patterns, unclassifiable patters or other HRCT findings). In five studies, patients were classified into two groups: UIP and non-UIP. For the purposes of this analysis, the non-UIP group was classified under the “Other” category, as the original authors did not specify any further subclassification. The results are presented in Appendix A, Table A1.

Most studies were retrospective cohort studies, with sample sizes ranging from 20 to 250 patients. Quality assessment using NOS and JBI tools indicated that 16 studies were of low risk of bias, while 7 had moderate risk. Common limitations included retrospective design and heterogeneity in HRCT pattern definitions.

**Figure 2:**
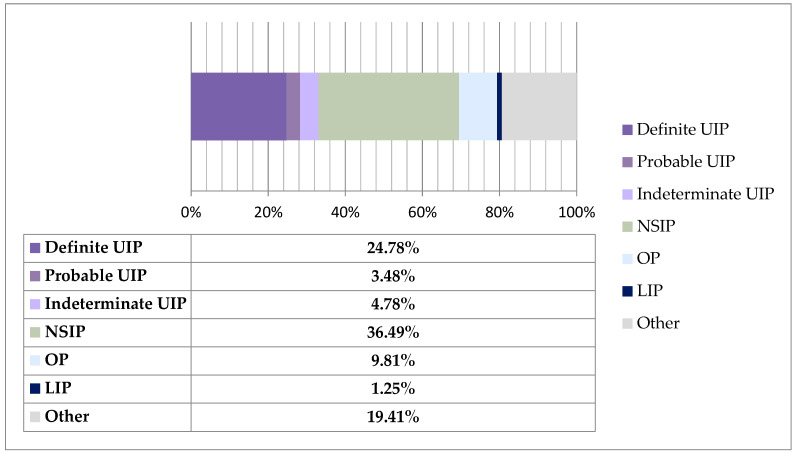
Proportion of HRCT patterns among CTD-ILD patients.

Figure 2 demonstrates the distribution of HRCT patterns among patients diagnosed with CTD-ILD. The bar chart uses normalized values to show the contribution of each category relative to the total. The arithmetic mean was used for descriptive purposes only, despite the non-normal distribution of some of the data.

The proportions for each pattern are as follows: Definite UIP—24.78%, Probable UIP—3.48%, Indeterminate for UIP—4.78%, NSIP—36.49%, OP—9.81%, LIP—1.25%, and Other—19.41%. Therefore, we can conclude that NSIP was the most frequently observed HRCT pattern among these patients, which is consistent with findings reported in the literature.

**Figure 3:**
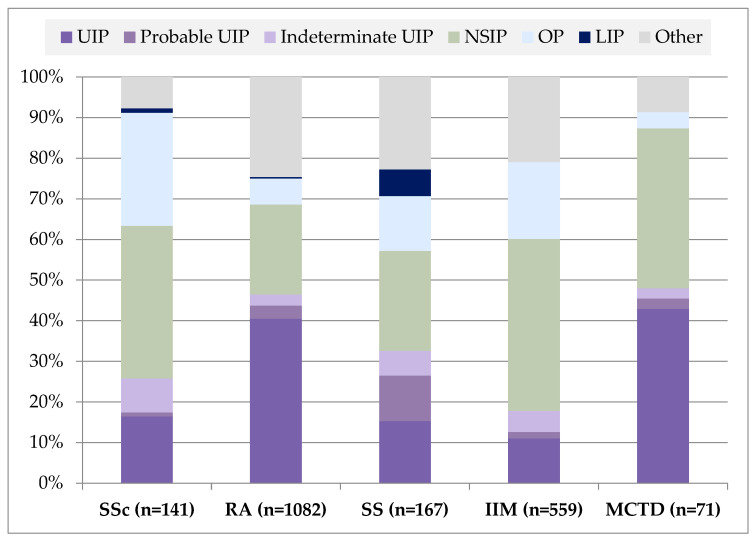
Distribution of ILD patterns across CTDs.

Figure 3 illustrates the distribution of HRCT-ILD patterns across various CTDs, as identified through a comprehensive literature review. The most prevalent pattern varies significantly depending on the underlying CTD. In SSc, NSIP is the predominant pattern, accounting for approximately 45–50% of cases, followed by probable UIP and indeterminate UIP. In contrast, RA demonstrates a higher prevalence of UIP and probable UIP patterns, together comprising the majority of cases—around 60%—with NSIP and other patterns being less common. For SS, the distribution is more heterogeneous, with NSIP and LIP showing substantial representation, along with a notable proportion of patients having an indeterminate pattern or other rare forms. IIMs are most frequently associated with NSIP (around 50%), followed by OP and UIP-related patterns. In MCTD, NSIP also predominates, making up nearly half of the cases, accompanied by a relatively high proportion of probable UIP and indeterminate for UIP. Across all CTDs, patterns such as LIP and OP appear more sporadically and are generally less common, though LIP is particularly more represented in SS.

Due to the limited number of studies and small sample sizes, formal investigations of heterogeneity, such as subgroup analyses or meta-regression, were not achievable and sensitivity analyses could not be conducted to assess the robustness of the results. Similarly, formal assessment of reporting bias (e.g., funnel plots, Egger’s test) could not be performed, and therefore, the risk of bias arising from missing results cannot be excluded.

Due to the non-Gaussian distribution, non-parametric tests were used for group comparisons. The non-parametric Kruskal–Wallis test revealed statistically significant differences in the distribution of HRCT patterns among CTD-ILDs. For the UIP pattern, the test showed a highly statistically significant difference across CTD subtypes (*p* < 0.001). Post hoc Dunn’s test demonstrated that patients with rheumatoid arthritis had a significantly higher prevalence of the UIP pattern compared to those with idiopathic inflammatory myopathies. Regarding the NSIP pattern, the Kruskal–Wallis test also indicated a statistically significant difference among CTDs (*p* < 0.05); however, Dunn’s post hoc analysis did not identify any significant pairwise differences between specific disease groups after adjustment for multiple comparisons. This suggests that while a global difference exists, individual group differences may be subtle or influenced by sample size limitations. For the LIP pattern, a statistically significant difference was observed (*p* < 0.05), and post hoc analysis revealed that patients with Sjögren’s syndrome had a significantly higher prevalence of LIP compared to those with idiopathic inflammatory myopathies and mixed connective tissue disease. All statistical analyses described above, along with the relevant data, are presented in Table 2.

These results highlight the variability of ILD manifestations across various CTD subtypes and imply that certain CTDs demonstrate a predilection for distinct radiological profiles. The heightened incidence of the UIP pattern in rheumatoid arthritis corresponds to its recognized poorer prognosis, ILD being among the most serious and potentially fatal complications of rheumatoid arthritis, increasing mortality threefold [8]. Meanwhile, the prevalence of LIP in Sjögren’s syndrome may indicate the underlying lymphocytic infiltration that characterizes this disease. On the other hand, NSIP was observed in all CTD subgroups; the lack of significant pairwise differences may suggest a more generalized or shared inflammatory pathophysiology.

The overall certainty of the evidence was considered low to very low for all outcomes, mainly due to the small number of studies, methodological heterogeneity, and moderate risk of bias identified in several included studies. Therefore, the findings should be interpreted with caution and regarded as exploratory rather than definitive estimates.

**Figure 4:**
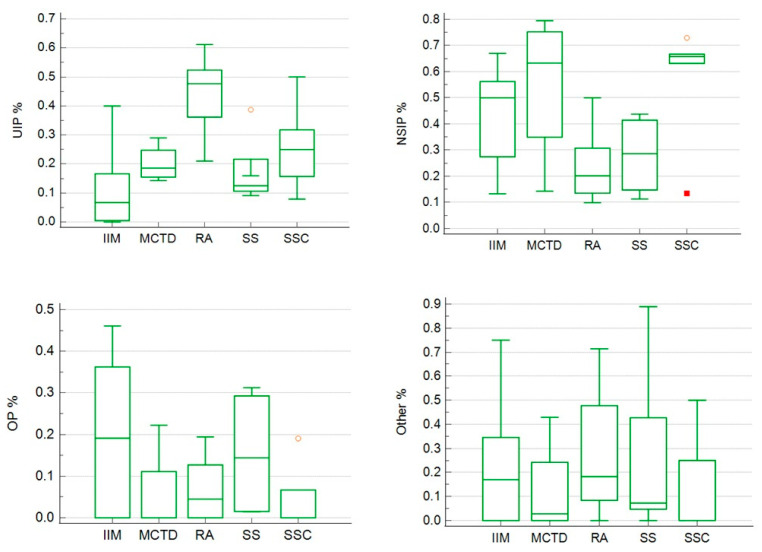
Comparative distribution of ILD patterns (UIP, NSIP, OP, and Other) across CTDs: box-and-whisker analysis. Orange circle: outlier (value beyond 1.5 × the interquartile range), red square: extreme outlier (value beyond 3 × the interquartile range).

Figure 4 presents a box-and-whisker analysis of ILD pattern distribution across CTDs, highlighting differences between groups.

### 3.3. HRCT Imaging Patterns in Connective Tissue Disorders

HRCT is the gold standard tool for identifying interstitial abnormalities in ILDs, which are often missed by plain chest radiography. It offers a more sensitive evaluation of lung involvement, particularly in diseases like CTD-ILDs, by producing high-resolution images that help identify specific patterns associated with ILD, such as non-specific interstitial pneumonia (NSIP), usual interstitial pneumonia (UIP), organizing pneumonia (OP) and lymphoid interstitial pneumonia (LIP) [1,9]. By combining HRCT findings with pulmonary function tests (PFT) and clinical assessments through a multidisciplinary approach, we can establish a definitive diagnosis, evaluate disease severity and determine the most appropriate subsequent management strategy [10].

**Figure 5:**
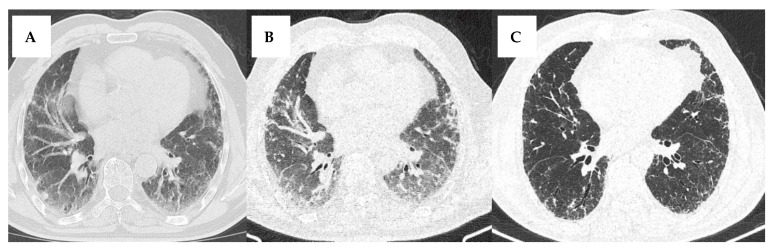
Impact of HRCT Acquisition Parameters on the Assessment of ILD: Comparison of Slice Thickness and Respiratory Phase. We present the case of a 71-year-old man who was evaluated in our clinic for a three-month history of dry cough and exertional dyspnea on moderate effort. He had previously undergone two imaging assessments in different centers, performed eight days apart. The first scan (**A**) was acquired in expiration, with a slice thickness of 2 mm. The second scan had a slice thickness of 0.6 mm and included images captured in both expiration (**B**) and inspiration (**C**). Findings include diffuse interstitial lung abnormalities with reticulation, ground-glass opacities, and traction bronchiectasis. When comparing the first two images (both acquired in expiration), the second image appears to have more defined lung structures. The 2 mm scan has a slightly more blurred appearance, reducing the visibility of small airway or parenchymal abnormalities. Furthermore, on the third image (acquired in inspiration), the lungs are more inflated, allowing better differentiation of true ground-glass opacities versus normal lung attenuation. In addition, airway structures appear less crowded, making it easier to assess traction bronchiectasis and the reticular pattern.

The use of HRCT requires specific technical considerations, including thin slice thickness (0.5–1.5 mm) and high spatial frequency reconstruction algorithms. Improper settings can result in the misinterpretation of ILD-related abnormalities [9] (Figure 5). The protocol should include the reconstruction of images in axial, coronal, and sagittal planes to provide comprehensive views of lung structures [11]. Images should be acquired during full inspiration, whereas the expiratory phase is sometimes recommended for detecting air trapping in small airway diseases [12]. Prone positioning can help differentiate between dependent atelectasis and early fibrosis by altering the distribution of lung densities. In cases of dependent atelectasis, the lung regions affected typically improve in appearance when the patient is placed in a prone position, as the atelectatic areas may re-expand [13].

The HRCT patterns are presented here in order of clinical relevance and illustrative purposes (UIP, NSIP, LIP, OP). While NSIP is generally the most frequent pattern in CTD-ILDs, UIP is presented first to highlight its distinctive imaging features and prognostic implications. It should be noted that the onset and evolution of these patterns may vary among individual patients, and some patterns, such as OP, can precede fibrotic changes like NSIP or UIP.

#### 3.3.1. UIP Pattern

Usual Interstitial Pneumonia (UIP) is a specific pattern of lung disease that is often associated with idiopathic pulmonary fibrosis (IPF). The presence of a radiologic UIP pattern is highly predictive of a histopathological UIP pattern, especially in the appropriate clinical context [12]. Currently, the reliable imaging features of UIP are considered diagnostic, eliminating the need for a surgical lung biopsy [14,15].

**Figure 6:**
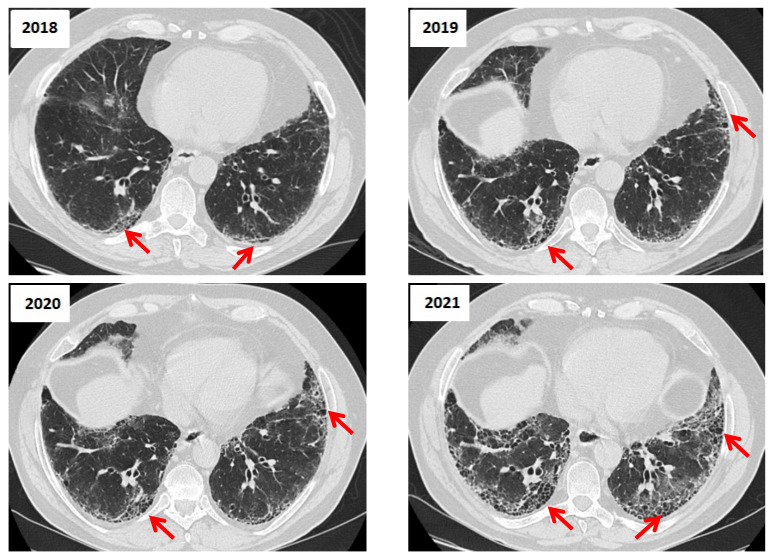
Longitudinal HRCT Changes of a UIP Pattern in a Patient With IPF (2018–2021). 2018: The baseline HRCT shows subpleural and basal predominant reticulation with early honeycombing and traction bronchiectasis, consistent with UIP. Ground-glass opacities are minimal (red arrows). 2019: There is a slight increase in reticular opacities and honeycombing, with more pronounced architectural distortion, suggesting disease progression (red arrows). 2020: Further fibrotic progression is evident, with an increase in honeycombing and traction bronchiectasis. The lung volume appears slightly reduced (red arrows). 2021: The final HRCT shows extensive fibrosis with coalescent honeycombing, more severe traction bronchiectasis and significant volume loss, indicative of advanced disease. Ground-glass opacities remain minimal, supporting a fibrotic rather than an inflammatory process (red arrows).

According to the updated ATS/ERS/JRS/ALAT guideline by Raghu G et al. (Am J Respir Crit Care Med, 2022), the HRCT patterns can be classified as UIP, probable UIP, indeterminate UIP, or suggestive of an alternative diagnosis [14]. The difference between UIP and probable UIP is based on the specific imaging features. A definitive UIP pattern is characterized by basal and subpleural predominant reticular pattern with honeycombing, with or without traction bronchiectasis and mild ground-glass opacities (GGO) (Figure 6).

**Figure 7:**
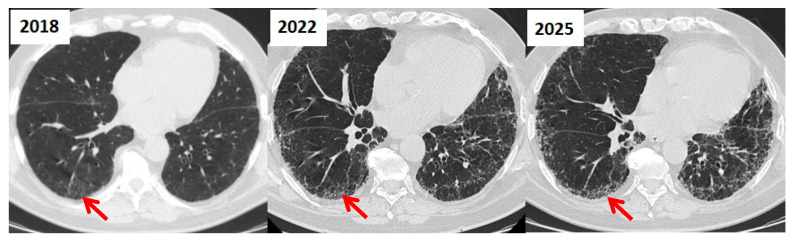
Evolution From Probable UIP to Definite UIP on HRCT in a Patient with IPF over Seven Years (2018–2025). Serial HRCT scans from 2018, 2022, and 2025 show progressive fibrotic changes consistent with idiopathic pulmonary fibrosis. The 2018 scan demonstrates a probable UIP pattern with subpleural, basal reticulations and traction bronchiectasis, but no definitive honeycombing (red arrow). By 2022, early honeycombing appears alongside increased fibrosis (red arrow). In 2025, there is extensive subpleural honeycombing, architectural distortion, and traction bronchiectasis, meeting criteria for a typical UIP pattern (red arrow). This evolution illustrates radiologic progression from a probable to a typical UIP pattern over time.

In contrast, a probable UIP pattern lacks honeycombing but retains the same distribution, the presence of reticular abnormalities with traction bronchiectasis or bronchiolectasis and absence of subpleural sparing (Figure 7). An indeterminate UIP pattern refers to cases where the imaging findings do not confidently fit into either the definite UIP or probable UIP categories, yet they also lack definitive features of an alternative diagnosis. This pattern typically includes fibrosis with an unclear distribution, subtle reticulation, or atypical features that prevent classification as UIP or probable UIP. In some cases, the imaging may show features suggestive of fibrosis but without sufficient specificity to confidently suggest a UIP pattern [16]. Given the diagnostic uncertainty, patients with an indeterminate UIP pattern often require additional clinical correlation, multidisciplinary discussion, or, in selected cases, lung biopsy for further characterization [14].

#### 3.3.2. NSIP Pattern

NSIP (Non-Specific Interstitial Pneumonia) is a type of ILD characterized by specific histological and radiological features. In the literature, NSIP patterns have been observed in various cases, with some classified as related to underlying CTDs and others as idiopathic NSIP (iNSIP) without a definitive cause [17].

Histologically, NSIP is characterized by both temporal and spatial uniformity within biopsy specimens, in contrast to the pronounced heterogeneity observed in UIP patterns. This uniformity facilitates the categorization of NSIP as a distinct pathological entity. It can be further categorized into subtypes, “fibrotic NSIP” and “cellular NSIP,” based on the predominant histological characteristics observed in lung biopsies [12].

**Figure 8:**
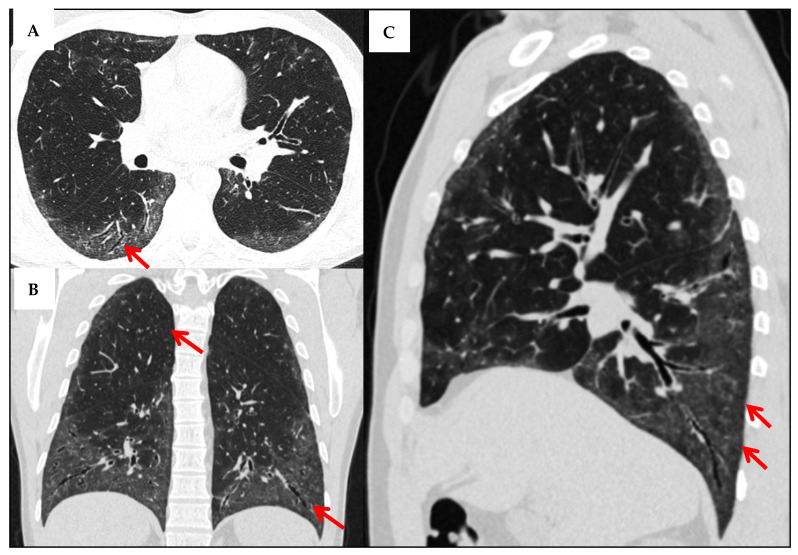
Multiplanar HRCT Evaluation in a 43-Year-Old Patient with Systemic Sclerosis. The axial slice (**A**) demonstrates bilateral, predominantly peripheral and basal-predominant reticular opacities with associated traction bronchiectasis (red arrow). There is no evident honeycombing or significant ground-glass opacity. These features are consistent with a fibrotic NSIP pattern, which is the most common form of ILD in systemic sclerosis. In the coronal view (**B**), the upper lobes appear relatively spared, supporting the diagnosis of an NSIP pattern (first red arrow). Traction bronchiectasis is also evident (second red arrow). The sagittal section (**C**) illustrates vertical extent and subpleural fibrosis with traction bronchiectasis, most prominent in the posterior basal segments. Also, there appears to be relative sparing of the immediate subpleural region (red arrows).

The NSIP pattern on HRCT is characterized by ground-glass opacities, indicating inflammation or partial airspace filling, along with a reticular pattern reflecting interstitial fibrosis. The abnormalities predominantly affect the peribronchovascular interstitium of the lower lobes and often exhibit subpleural sparing, distinguishing NSIP from UIP. Unlike UIP, NSIP typically lacks honeycombing but may present with traction bronchiectasis due to surrounding fibrosis (Figure 8) [18]. A multidisciplinary discussion involving radiologists, pulmonologists, and pathologists is often necessary to arrive at a consensus diagnosis based on HRCT findings [18].

#### 3.3.3. OP Pattern

Organizing pneumonia (OP) is a clinicopathologic entity that represents a nonspecific response to lung injury, characterized histologically by plugs of granulation tissue filling the alveoli, alveolar ducts, and sometimes bronchioles, while preserving the underlying lung architecture. OP can occur as a primary idiopathic condition known as cryptogenic organizing pneumonia (COP), or as a secondary process associated with various triggers including infections, autoimmune diseases, medications, radiation, and aspiration [19].

**Figure 9:**
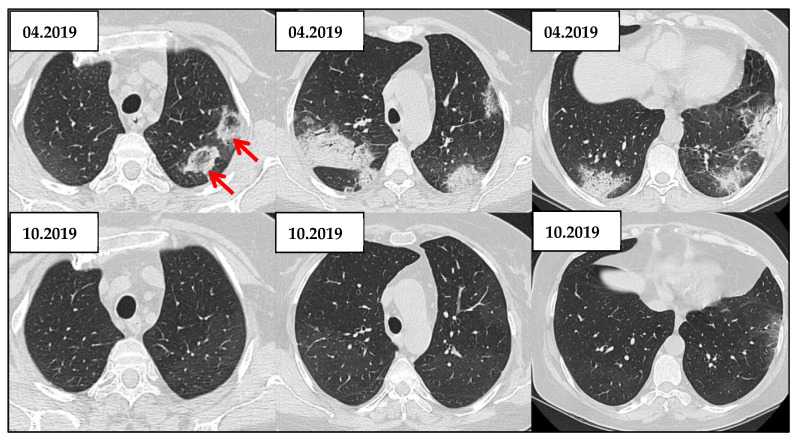
Radiologic Evolution of OP Under Corticosteroid Therapy. This case illustrates the radiological evolution of a 60-year-old woman over a six-month period, from April 2019 (top row) to October 2019 (bottom row). Initial scans reveal bilateral, asymmetric consolidations in peripheral and peribronchovascular regions. There are several focal areas where rounded regions of ground-glass opacities are surrounded by a rim of consolidation, creating the characteristic atoll appearance (red arrow), suggesting the OP diagnosis. In contrast, the follow-up images from October 2019 demonstrate a marked radiologic improvement, with near-complete resolution of the previously noted consolidations and ground-glass opacities, indicating a favorable response to corticosteroid therapy, additionally supporting the diagnosis of OP.

Radiologically, OP in CTD-ILD often mimics COP and the HRCT reveals a wide spectrum of findings. The most typical pattern consists of bilateral, peripheral and peribronchovascular consolidations, often in the lower lobes. These may show air bronchograms and demonstrate a migratory behavior over time. Ground-glass opacities (GGOs), perilobular opacities, and the reversed halo sign (atoll sign) are also commonly seen (Figure 9). Nodules, parenchymal bands, and linear or reticular opacities may also appear [3]. However, in CTDs, OP may coexist with reticular opacities, traction bronchiectasis, and even early fibrosis—making imaging interpretation more complex [20].

Histopathologically, OP is defined by intraluminal buds of granulation tissue—termed Masson bodies—composed of fibroblasts embedded in a myxoid matrix, within the alveolar spaces and ducts. These Masson bodies are surrounded by relatively normal lung parenchyma, with only mild interstitial inflammation. In contrast to fibrosing interstitial pneumonias like UIP, there is no architectural distortion or significant collagen deposition. In rare cases, OP may evolve into chronic fibrosis, sometimes overlapping with patterns such as NSIP or showing features like traction bronchiectasis and subpleural reticulation [20].

Diagnosis is based on clinical–radiologic correlation, and sometimes tissue biopsy, is required to exclude alternative diagnoses. While transbronchial biopsy or cryobiopsy may suffice, surgical lung biopsy is rarely necessary if the clinical and imaging profile is typical. A multidisciplinary approach involving pulmonologists, radiologists, and pathologists is often needed to establish a confident diagnosis [19,20].

From a clinical perspective, the presence of an OP pattern in CTD-ILD is typically associated with a better prognosis compared to fibrosing patterns like UIP. It often responds well to corticosteroids, and patients may experience significant radiologic and symptomatic improvement. However, relapses are possible—especially during tapering of steroids—and some patients may eventually develop chronic fibrosing disease, including NSIP or fibrotic progression within the OP spectrum.

In conclusion, recognizing this pattern is important because it has therapeutic relevance, since OP is highly steroid-responsive, whereas fibrotic NSIP or UIP may require immunosuppressive or antifibrotic therapies [19].

#### 3.3.4. LIP Pattern

Lymphocytic interstitial pneumonia (LIP) is a rare interstitial lung disease that occurs in the context of immune dysregulation, most frequently associated with CTDs like primary Sjögren’s syndrome (pSS). It is characterized by diffuse infiltration of the alveolar septa and interstitium by lymphocytes and plasma cells, often accompanied by reactive lymphoid follicles. This histological picture reflects a benign, polyclonal lymphoid proliferation rather than a malignant process, though it may be challenging to distinguish clinically and radiologically from low-grade lymphomas such as mucosa-associated lymphoid tissue (MALT) lymphoma [21]. In comparison to malignant lymphoproliferative disorders, LIP tends to show a more diffuse but less destructive pattern. Nonetheless, biopsy is often required to confirm its benign nature, especially when radiologic findings include larger nodules, consolidations, or pleural effusions—features more typical of MALT lymphoma [22].

**Figure 10:**
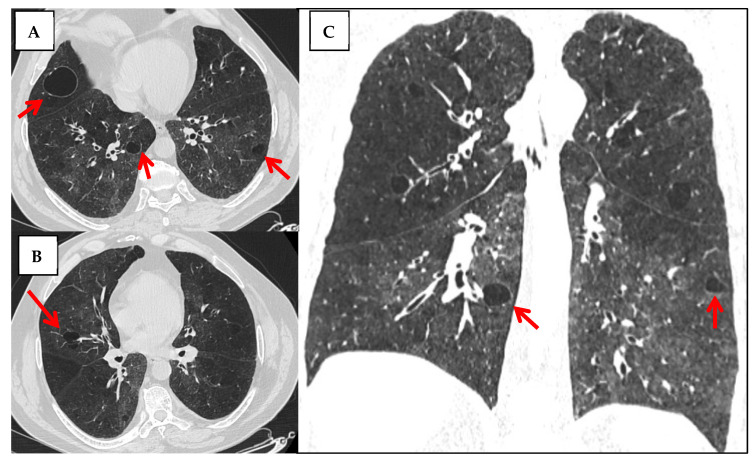
HRCT Features Suggestive of Lymphocytic Interstitial Pneumonia in a Patient with Suspected Autoimmune Disease. Axial (**A**,**B**) and coronal (**C**) views show multiple thin-walled cysts with peribronchovascular and subpleural distribution, predominantly in the lower lobes (red arrows). Patchy areas of ground-glass opacity are seen throughout both lungs, along with ill-defined centrilobular and subpleural nodules. Subtle peribronchovascular interstitial thickening is also noted. This constellation of findings—diffuse ground-glass opacities, cystic change, nodularity, and perilymphatic distribution—is highly suggestive of LIP, particularly in the clinical context of autoimmune disease such as Sjögren’s syndrome.

On HRCT, typical imaging features include widespread ground-glass opacities, small centrilobular or subpleural nodules, and numerous thin-walled cysts, particularly in peribronchovascular distribution. Peribronchovascular thickening and mild interlobular septal thickening may also be observed [23]. These findings can lead to areas of uneven lung attenuation due to air-trapping, especially when lymphocytic bronchiolitis is also present (Figure 10) [1].

Table 3 summarizes the radiologic features of common ILD patterns, including UIP, NSIP, OP, and LIP.

### 3.4. Connective Tissue Disorders

#### 3.4.1. Systemic Sclerosis

Systemic sclerosis (SSc) is a multifaceted autoimmune rheumatic disease characterized by a combination of pathological features. These include: vascular dysfunction, immune system dysregulation, and progressive fibrosis of the skin and the internal organs [24]. The vasculopathy begins early with endothelial cell injury, leading to luminal narrowing and eventually obliteration, inducing tissue ischemia. Simultaneously, because of a disfunction of the immune system, the production of autoantibodies and pro-fibrotic cytokines begins. The most notable one is transforming growth factor-beta (TGF-β), which is pivotal in the fibrotic cascade. This cytokine stimulates fibroblast activation and their differentiation into myofibroblasts. This results in excessive collagen and extracellular matrix deposition in the skin and visceral organs [25].

It is considered a rare disease: the occurrence rate is roughly 10 cases per million individuals annually [1]. It predominantly affects females in a 4:1 ratio, often manifesting between the ages of 30 and 70 [26]. Epidemiologic data further indicate that SSc-associated ILD is the leading cause of death in this population, representing approximately one-third of all mortality attributable to disease [26]. This accentuates the imperative necessity for prompt identification, rigorous observation and action. A Canadian study estimates that ILD occurs in about 64% (of 289 patients) when assessed using HRCT, and in only 22% when chest X-ray is used, highlighting the sensitivity of HRCT [27].

The possible lack of clinical manifestations in the initial or less severe stages of the disease can delay the diagnosis of SSc-ILD, as patients may remain asymptomatic despite ongoing pulmonary involvement [10]. Later on, the most prevalent pulmonary symptom is exertional dyspnea, accompanied by a persistent dry cough and inspiratory “velcro” crackles on auscultation, that are not attributable to alternative etiologies. Physiologically, patients often exhibit a restrictive ventilatory pattern and decreased diffusion capacity for carbon monoxide (DLCO) [1]. Risk factors for developing ILD in SSc include anti-SCL-70 and speckled antinuclear antibodies positivity, anti-TRIM21 positivity, male gender, African American race, diffuse disease form, recent disease onset (within the first 5–7 years), and elevated acute phase reactants [28].

Recent global recommendations endorse the utilization of baseline HRCT for all patients recently diagnosed with systemic sclerosis, as it provides enhanced sensitivity in identifying early interstitial lung disease, even in the absence of clinical manifestations. This methodology is corroborated by the 2024 guideline from the British Society for Rheumatology, which emphasize the significance of HRCT in the early identification of ILD and the formulation of individualized management strategies [29]. Furthermore, expert consensus elucidates that pulmonary function tests in isolation may overlook early-stage ILD, thus accentuating the necessity of incorporating HRCT during the diagnostic process [30]. Moreover, in the SENCIS trial, despite significant pulmonary fibrosis confirmed by HRCT and a measurable decline in lung function, 20% of participants reported no cough, and 30% did not experience any dyspnea [31].

**Figure 11:**
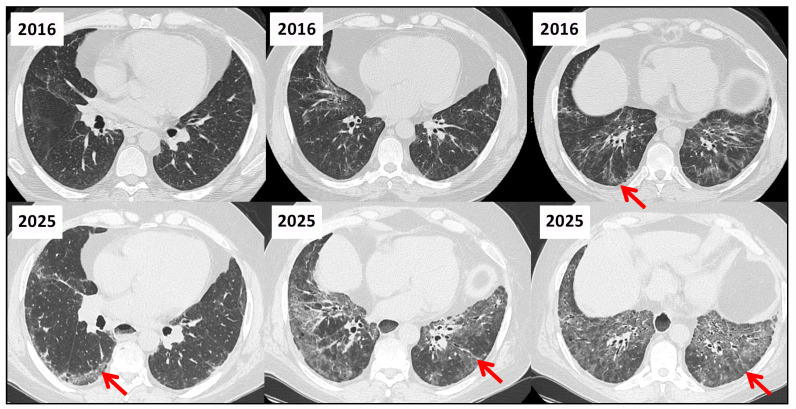
Serial HRCT Scans in a 53-Year-Old Patient with Systemic Sclerosis and ILD with a NSIP Pattern (2016–2025). Axial HRCT images acquired in 2016 (top row) and 2025 (bottom row) demonstrate radiological progression of fibrotic NSIP in a patient with systemic sclerosis. In 2016, the scans reveal bilateral, symmetric reticular opacities with peripheral and basal predominance, mild traction bronchiectasis, and relative subpleural sparing—features consistent with fibrotic NSIP. Nine years later, the 2025 images show a marked increase in reticulation and traction bronchiectasis (red arrows). There is visible lower lobe volume loss and architectural distortion, indicating progression of fibrotic remodeling. These findings reflect a worsening fibrotic phenotype over time, emphasizing the chronic and progressive nature of SSc-associated ILD. Mild to moderate dilatation of the esophagus is visible, more prominently in the 2025 images, consistent with progressive esophageal involvement typical of systemic sclerosis. The presence of intraluminal gas and increased luminal diameter suggests dysmotility, which may contribute to chronic aspiration and exacerbate pulmonary disease progression in SSc.

The most common radiological pattern reported by Joy et al. is NSIP, observed in approximately 75% of cases, typically characterized by ground-glass opacities and reticular changes with a lower lobe predominance (Figure 11), while UIP is less frequent, noted in around 20% of cases [32]. In our review, NSIP also emerged as the predominant pattern, accounting for approximately 45–50% of cases, followed by probable UIP and indeterminate UIP patterns, which together represent about 25% of cases. This distribution is consistent with previous findings, but with some variability likely due to differences in study populations. UIP pattern carries a worse prognosis and it is associated with more severe fibrosis, traction bronchiectasis, and honeycombing. In contrast to previous reports where other HRCT patterns accounted for roughly 5% of cases, in our review organizing pneumonia (OP) was observed in approximately 30% of patients [32]. The extent and pattern of fibrosis seen on HRCT are strong predictors of disease progression and mortality. The use of semi-quantitative scoring systems such as the Warrick score enables more accurate and reproducible assessments of disease extent. Several studies have shown that increasing HRCT scores correlate inversely with pulmonary function test results, confirming HRCT’s value in evaluating severity [33].

While ILD continues to be the primary pulmonary expression of SSc, other respiratory complications markedly contribute to the overall disease burden and influence prognosis. These include pulmonary arterial hypertension (PAH), esophageal dysfunction with aspiration events and lung cancer. PAH constitutes one of the most critical complications associated with SSc. Among the spectrum of CTDs, patients diagnosed with SSc exhibit the highest probability for the development of PAH, with prevalence estimates reaching as high as 15%. Factors that elevate the risk of PAH in individuals with SSc include advanced age, prolonged disease duration, reduced DLCO in the absence of substantial PFT impairment (FVC/DLCO > 1.6 in ILD), elevated levels of brain natriuretic peptide (BNP) or N-terminal pro brain natriuretic peptide (NT-proBNP), increased uric acid levels, limited cutaneous SSc subtype (lcSSc), as well as the presence of anti-centromere antibodies and telangiectasias [34,35]. PAH in the context of SSc is generally characterized as a manifestation of Group 1 pulmonary hypertension, arising from a complex interaction involving endothelial dysfunction, immune system activation, and fibroproliferative alterations within the pulmonary vasculature. Furthermore, SSc patients may also experience Group 3 pulmonary hypertension (attributable to interstitial lung disease) in cases where significant pulmonary fibrosis is present. Right heart catheterization (RHC) is acknowledged as the definitive diagnostic procedure, establishing a mean pulmonary arterial pressure (mPAP) exceeding 20 mmHg, pulmonary arterial wedge pressure (PAWP) of 15 mmHg or less, and pulmonary vascular resistance (PVR) surpassing 2 Wood units [34]. The DETECT algorithm serves as a validated screening tool tailored for SSc patients with disease duration exceeding 3 years and DLCO below 60%. This instrument integrates clinical parameters, serological data, and echocardiographic indicators to identify patients who require RHC [36]. On HRCT, a main pulmonary artery diameter of 29 mm or greater in the presence of ILD exhibits a robust association with the hemodynamic diagnosis of pulmonary hypertension. Additional indicators include a pulmonary artery to aorta ratio exceeding 1 (PA/A > 1) and the presence of dilated right heart chambers [35].

The gastrointestinal system is affected in approximately 90% of individuals afflicted with SSc. Furthermore, involvement of the esophagus serves as an initial indicator of the pathology, which can be identified through the observation of an expanded esophageal lumen on HRCT, measuring between 1.2 and 4 cm, with a mean of 2.3 cm and containing either gas or fluid [10,37]. Aspiration pneumonia arises as a consequence of esophageal involvement that is secondary to the underlying pathological condition [37].

Several analyses have demonstrated that the rate of neoplastic conditions, particularly affecting the respiratory system, is pronounced among those identified with SSc. The incidence has been established to peak at 10.7%, with SSc smokers having a risk of developing lung carcinoma that is increased sevenfold compared to non-SSc smoking counterparts [37].

Table 4 provides a summary of features of SSc-ILD discussed above.

#### 3.4.2. Rheumatoid Arthritis

Rheumatoid arthritis (RA) is a systemic autoimmune condition. The global incidence of RA is estimated to range from 0.5 to 1% of the adult population, and it is more common in women, with a female-to-male ratio of about 3:1 [38]. Is a condition that influences the immune response of the body and mainly targets the joints that are lined with synovial membranes; nonetheless, it can also present in various extra-articular organs. In the pulmonary system, these manifestations can involve multiple lung compartments, including both the major and minor airways, interstitial spaces, pleural membranes, and pulmonary vasculature, leading to an array of radiological presentations, such as ILD, nodular formations or masses, bronchiectasis, pleural effusions, alongside manifestations indicative of pulmonary hypertension [39].

Interstitial lung disease is among the most serious and potentially fatal complications. Data suggest that close to 10% of RA patients might have clinically observable ILD, while subclinical indications could be identified in as many as 30% when sensitive imaging modalities, like HRCT, are utilized [40]. The presence of ILD significantly worsens the prognosis of RA, with affected individuals having a threefold increase in mortality compared to those without interstitial lung involvement [8].

The relationships that link rheumatoid arthritis to interstitial lung disease are not fully understood; nevertheless, they seem to involve a complex interaction of genetic vulnerability, environmental contributors, immune system disturbances, and atypical tissue repair mechanisms. A significant immunopathological occurrence is citrullination, signifying the transformation of arginine residues into citrulline, which causes the generation of neoantigens identifiable by anti-citrullinated peptide antibodies (ACPAs). A vital function of these antibodies is seen in the pathology of RA, showing connections to inflammatory responses in both the joints and the pulmonary system [8]. The occurrence of RA-ILD demonstrates an upward trend with age, while male gender is associated with an increased risk [41]. Other important risk contributors consist of previous smoking habits, high disease activity, and positive serological markers including rheumatoid factor (RF) and ACPAs [34]. Furthermore, a documented history of emphysema serves as an independent prognostic factor for increased mortality [41].

Cigarette smoking is not only a risk factor for RA development but is also associated with more severe lung involvement. Beyond its recognized influence in the progression of seropositive RA, smoking significantly impacts pulmonary immune tolerance by enhancing protein citrullination via the increased activity of peptidylarginine deiminase enzymes. This biochemical process promotes the localized production of ACPAs. On a microscopic scale, the components of cigarette smoke inflict injury on epithelial and endothelial layers, consequently nurturing a supportive condition for chronic inflammation, fibroblast engagement and extracellular matrix deposition. These pathological alterations are fundamental to the fibrotic remodeling that characterizes various presentations of RA-ILD. Clinically and radiologically, individuals with RA who smoke are more prone to display imaging patterns indicative of UIP, often in conjunction with emphysematous alterations, alongside exhibiting more extensive airway pathologies such as bronchiolitis and bronchiectasis [42].

Patients with RA-ILD may show diverse nonspecific respiratory symptoms, which can include difficulty breathing during exertion, a nonproductive cough, or a decreased ability to undertake physical activities. Clinical examination frequently reveals the presence of bilateral basal inspiratory crackles, and in more severe instances, indicators of respiratory failure may be observed. A subset of patients may receive an incidental diagnosis through HRCT scans conducted for alternative clinical indications [28]. In some cases, ILD may precede joint involvement, complicating the diagnostic process. The clinical course of RA-ILD is highly variable. Some individuals remain stable for years, while others develop rapidly progressive disease that mimics IPF [43].

There is growing consensus regarding the application of risk-based methodologies for HRCT screening and monitoring in individuals diagnosed with RA, in contrast to SSc, where HRCT is advised at the baseline for all patients [29]. This discrepancy arises from the significantly higher prevalence of RA in comparison to SSc. Instead of adopting a standardized screening method for every RA patient, the optimal and cost-efficient strategy could be identifying those with a higher risk profile—older males with a smoking history, extended disease duration, and high levels of ACPAs or RF titers—for baseline HRCT and serial monitoring [42].

In patients with RA-ILD, the most frequently observed HRCT pattern is UIP. Across multiple cohorts, the UIP pattern has been identified in approximately 40% to 60% of patients. For example, a Finnish study by Nurmi et al. classified 59.3% of their RA-ILD cohort as having a UIP pattern, highlighting it as the dominant radiologic subtype [43]. Similarly, in our review, UIP was observed in approximately 45% of RA-ILD cases, reflecting a consistent prevalence within the reported range. In a different light, NSIP accounts for a smaller proportion, typically ranging from 15% to 25% in the literature, which aligns with our findings where NSIP was observed in approximately 20% of patients. OP was noted less frequently, representing a total of less than 20% of the cases, whereas in our review it accounted for approximately 10% of RA-ILD patients [43]. Mixed patterns (e.g., NSIP with organizing pneumonia or OP components) may also be seen. The UIP pattern is a strong negative prognostic marker, associated with accelerated decline and higher mortality, whereas the NSIP and other patterns generally confer a more favorable outlook [43].

**Figure 12:**
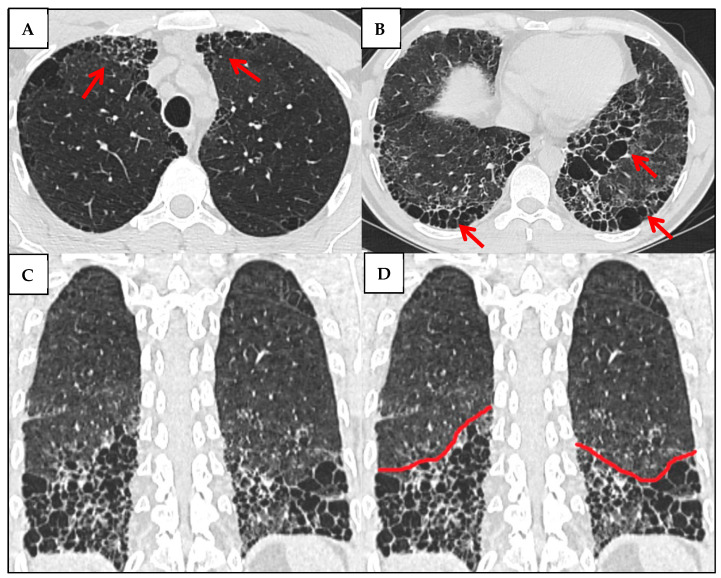
HRCT Scans in a 35-Year-Old Patient with Rheumatoid Arthritis and ILD with an UIP Pattern. Image (**A**) demonstrates the “upper lobe sign,” characterized by predominant fibrotic involvement of the anterior segments of the upper lobes (red arrows). Image (**B**) shows “exuberant honeycombing,” with extensive, clustered cystic airspaces comprising more than 70% of the fibrotic regions (red arrows). Coronal reconstructions (Images **C**,**D**) reveal the “straight edge sign,” particularly evident in Image D with the red demarcation line, indicating an abrupt interface between normal and fibrotic lung tissue, forming a well-defined linear boundary.

Importantly, certain HRCT features can help distinguish CTD-related UIP from IPF. These signs are significantly more common in CTD-associated ILDs, including RA, than in IPF, and their presence raises suspicion of an autoimmune etiology. However, they lack sensitivity and should be used in conjunction with serologic and clinical data [44]. Three signs have been proposed for this purpose. Anterior Upper Lobe (AUL) Sign, represents a concentration of fibrosis in the anterior segment of the upper lobes, with relative sparing of other areas. Straight-Edge (SE) Sign—presents as a sharp, linear demarcation between fibrotic and normal lung at the lung bases, without lateral extension, best appreciated on coronal images. Finally, Exuberant Honeycombing (EHC)—represents an extensive honeycomb cyst formation, comprising over 70% of the fibrotic regions [45] (Figure 12).

Individuals diagnosed with RA may also present pulmonary nodules in approximately 20% of cases, with dimensions ranging from 0.5 to 5 cm in diameter. Such manifestations are observed more frequently in male patients, smokers, those with subcutaneous nodules as well as elevated RF titers. Generally, these nodules are asymptomatic and are located peripherally within the mid and upper pulmonary zones. They possess the capacity to cavitate, enlarge, or spontaneously resolve. In atypical circumstances, these nodules may invade the pleural cavity, culminating in complications such as pneumothorax, pleural effusion, or empyema. The differential diagnosis must include nodules arising from infectious etiologies and neoplasms, thereby necessitating the possible implementation of bronchoscopy or biopsy for conclusive assessment [38].

In addition to the involvement of the lung parenchyma, RA exhibits a strong inclination to affect the small airways, thus significantly contributing to respiratory difficulties. The two main pathophysiological conditions linked to RA include constrictive bronchiolitis (CB), often called obliterative bronchiolitis, and follicular bronchiolitis (FB). Constrictive bronchiolitis (CB) is a fibrosing disorder characterized by bronchiolar narrowing and obliteration, emerging from fibrotic changes below the epithelium and associated inflammation. It constitutes the most severe manifestation within the small airways spectrum of RA and frequently results in progressive limitations in airflow. HRCT findings typically include: mosaic attenuation observed on inspiratory scans, air trapping discernible on expiratory images (most effectively visualized using paired inspiratory–expiratory HRCT), bronchial wall thickening, and centrilobular nodules. Follicular bronchiolitis (FB) is categorized as a lymphoproliferative disorder affecting the bronchioles, distinguished by reactive lymphoid hyperplasia localized around the small airways. HRCT findings associated with FB include centrilobular nodules, branching linear opacities (termed tree-in-bud), and ground-glass opacities manifesting in a peribronchial distribution. The distinction between CB and FB is important for both prognosis and therapeutic strategies, as CB is generally irreversible and may precipitate respiratory failure, whereas FB often demonstrates responsiveness to corticosteroid therapy [38].

Table 5 provides a summary of features of RA-ILD discussed above.

#### 3.4.3. Sjögren’s Syndrome

Sjögren syndrome (SS) represents a systemic autoimmune condition, ranking as the second most common disorder subsequent to rheumatoid arthritis. Recent assessments indicate that its incidence within Europe approximates 3.9 to 5.3 occurrences per 100,000 person-years. It exhibits marked female predominance (female-to-male ratio of 9–13:1), typically diagnosed around 56 years of age [46]. Pulmonary complications affect approximately 16% of patients, though subclinical abnormalities on HRCT are detected in up to 65% of asymptomatic individuals, emphasizing the importance of proactive screening [47].

This autoimmune condition primarily affects the exocrine glands, typically presenting with sicca symptoms—namely xerophthalmia and xerostomia—due to lymphocytic infiltration of the lacrimal and salivary glands. However, extra-glandular manifestations occur in approximately 30–40% of patients. Pulmonary involvement in primary Sjögren’s syndrome (pSS) can manifest in various forms, including upper and lower airway disease, interstitial lung disease (pSS-ILD), and, less commonly, lymphoproliferative disorders [21,48].

Several demographic, clinical and serologic variables have been identified as contributing factors to the development of ILD in individuals with Sjögren’s syndrome. These include advanced age, male sex, smoking history, higher ESSDAI (EULAR Sjögren’s syndrome disease activity index), prolonged disease course, coexistence of other autoimmune conditions, elevated inflammatory markers (erythrocyte sedimentation rate—ESR, C-reactive protein—CRP), signs of lymphocytic activation, such as autoantibody positivity (Anti-Ro/Ssa—especially Ro-52; Anti-La/SSb) or hypergammaglobulinemia [21,32,49].

The majority of patients afflicted with pSS-ILD typically exhibits pulmonary symptoms, including dyspnea, cough sometimes with sputum expectoration and thoracic discomfort. The severity of these symptoms can vary significantly, from asymptomatic to experiencing slight exertional dyspnea or, in more advanced situations, severe respiratory challenges [47].

The 2021 Consensus Guidelines for Evaluation and Management of Pulmonary Disease in Sjögren’s advocate for initial screening utilizing chest radiography and pulmonary function tests (PFT) for all individuals. In scenarios where pulmonary involvement is clinically presumed, HRCT is recommended as the diagnostic method of choice, for identifying pSS-ILD and other potential lymphoproliferative disorders such bronchus-associated lymphoid tissue (BALT) lymphoma, that are frequently overlooked in conventional chest X-ray assessments [47,49]. Patients with this disease exhibit a significantly high risk of developing B-cell lymphoma, estimated to be 15 to 20 times greater than that observed in the general population with a lifetime probability ranging from 5% to 10%. This high susceptibility is predominantly attributed to the chronic activation of B-cells. The majority of these malignancies consists mostly of B-cell non-Hodgkin lymphomas and they characteristically emerge in tissues that are subjected to inflammation associated with Sjögren’s syndrome [48].

**Figure 13:**
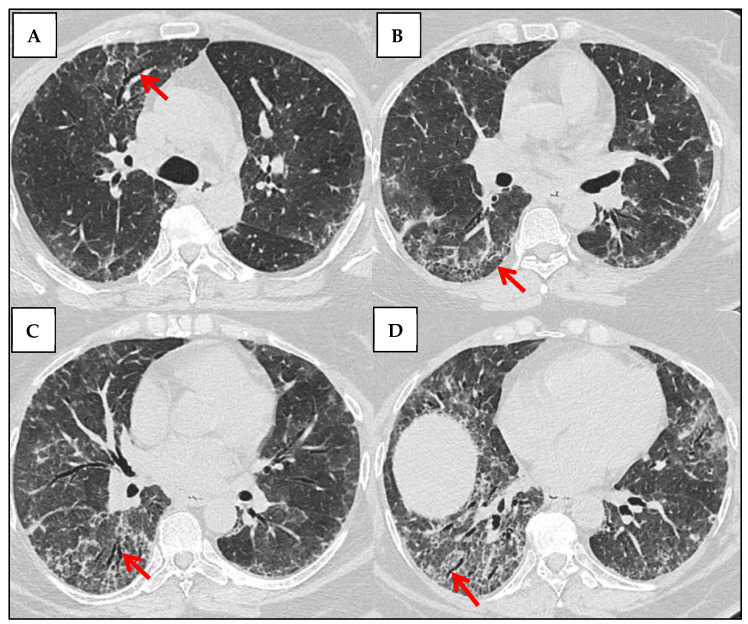
HRCT (2019) Demonstrating a Fibrotic NSIP Pattern in a Patient with Primary Sjögren’s Syndrome. A 49-year-old female with a 30 pack-year smoking history presented in February 2019 with progressive exertional dyspnea (mMRC grade 3) and significant resting hypoxemia (SpO_2_ 80% on room air). Serologic evaluation revealed positive anti-SSA (Ro) and anti-SSB (La) antibodies, accompanied by clinical manifestations of xerophthalmia and xerostomia, establishing the diagnosis of primary Sjögren’s syndrome. The axial HRCT images from 2019 demonstrate the characteristic radiological pattern of fibrotic NSIP. The images show bilateral, symmetric reticular opacities and ground-glass changes predominantly in the lower lung zones. There is clear subpleural sparing (**B**), visible as a thin rim of relatively preserved lung immediately beneath the pleura, which is a distinguishing feature of NSIP (red arrow). Architectural distortion and lower lobe volume loss are present, but there is no evidence of honeycombing. In addition to these findings, there is clear evidence of traction bronchiolectasis. This is seen as irregular, dilated bronchioles within areas of fibrotic lung parenchyma, reflecting traction on the small airways (**A**,**C**,**D**–red arrows).

Among patients with pSS-ILD, only 60% exhibited a single, distinct HRCT pattern. Within this subset, NSIP constituted the predominant manifestation, identified in 42% of instances (Figure 13), succeeded by UIP at 11%, and OP and LIP each at 4% [21]. Some studies have reported a higher prevalence of the LIP pattern, with rates reaching up to 17.4% [50]. In contrast, our review found a different distribution, with UIP observed in approximately 30% of cases, NSIP in about 25%, OP in 15%, and LIP in 5%, reflecting variability that may be influenced by differences in patient populations. Among patients exhibiting mixed HRCT patterns, the most commonly observed combinations included NSIP alongside features characteristic of OP or LIP [21]. Compared to other CTDs, cystic lung disease is more commonly seen in Sjögren’s syndrome. Frequently, it presents itself as a result of LIP. Nevertheless, the identification of cystic alterations, particularly when associated with pulmonary nodules, may also raise suspicion of underlying amyloidosis or BALT lymphoma [47]. Abnormalities affecting the bronchial and bronchiolar structures are also common in these patients, often presenting as centrilobular nodules and tree-in-bud patterns on HRCT, which reflect bronchiolar inflammation and peribronchiolar lymphoid infiltration. These imaging features, along with evidence of bronchiectasis, bronchial wall thickening, and signs of airflow obstruction such as air trapping and mosaic attenuation, point to significant small airways involvement. Such observations align with ongoing inflammatory mechanisms, including follicular bronchiolitis and constrictive bronchiolitis, and may play a role in the progression of the respiratory symptoms, despite the lack of significant interstitial alterations [46].

Table 6 provides a summary of features of pSS-ILD discussed above.

#### 3.4.4. Idiopathic Inflammatory Myopathies

Idiopathic inflammatory myopathies (IIMs), commonly referred to as myositis, are a group of disorders characterized by persistent inflammation and weakness of the skeletal musculature. These conditions frequently affect other organs, including the skin, heart, lungs and gastrointestinal tract. Traditionally, IIMs have been classified into polymyositis (PM), dermatomyositis (DM), and sporadic inclusion-body myositis (sIBM), based on their clinical presentation and histological features. Not long ago, two extra types, nonspecific myositis and immune-mediated necrotizing myopathy (IMNM), were identified [51]. Additionally, amyopathic dermatomyositis (ADM) is recognized as a variant of dermatomyositis characterized by the presence of classic cutaneous features, but with little or no clinical or laboratory evidence of muscle involvement [52].

Both DM and PM are rare disorders, exhibiting an annual incidence of less than 10 cases per million individuals [52]. They present significant similarities including proximal muscle weakness, elevated serum levels of muscle enzymes, electromyographic findings indicative of myopathy, the presence of inflammatory infiltrates within muscle tissue, and the detection of myositis-specific autoantibodies. Importantly, DM is also characterized by unique dermatological manifestations, such as Gottron’s papules, heliotrope rash, and mechanic’s hands [52].

Antisynthetase syndrome (ASyS) represents an uncommon and clinically diverse systemic autoimmune condition that falls under the category of IIM. This condition is marked by the existence of one or more of the following clinical features: ILD, myositis, non-erosive seronegative arthritis, Raynaud’s phenomenon, mechanic’s hands and persistent fever without a clear cause [53]. These symptoms are typically associated with autoantibodies targeting aminoacyl-transfer RNA synthetases (anti-ARS antibodies). These include Anti-Jo-1, Anti-PL-7, Anti-PL-12, Anti-OJ, Anti-EJ, Anti-KS, Anti-ZO and Anti-Ha [54]. ILD is present in approximately 79% to 95% of cases and may appear before the onset of muscle involvement in up to half of patients [51]. Notably, patients with antisynthetase syndrome who have anti-PL7 or anti-PL12 antibodies tend to exhibit more severe lung involvement and demonstrate lower baseline forced vital capacity (FVC) and DLCO compared to those with anti-Jo-1 antibodies [55]. Rapidly progressive interstitial lung disease (RP-ILD) constitutes a particularly critical and life-threatening complication in individuals diagnosed with IIMs. Two principal categories of myositis-specific autoantibodies (MSAs) that correlate with ILD are anti-melanoma differentiation-associated gene 5 (anti-MDA5) antibodies and anti-ARS antibodies. RP-ILD exhibits a strong association with the presence of anti-MDA5 antibodies, whereas anti-ARS antibodies are intermittently linked to rapidly progressive variants of ILD in these patients [56]. Remarkably, patients with anti-MDA5 antibodies may also present with distinctive skin manifestations, such as cutaneous ulcerations and painful palmar papules. Around half of those who test positive for anti-MDA5 do not exhibit muscle weakness, often associated with ADM [55].

Furthermore, advanced age is recognized as a significant risk factor for the onset of ILD among individuals diagnosed with IIMs. The presence of elevated inflammatory markers, including ESR and CRP, correlates with an increased probability of developing ILD. Moreover, the identification of black race as a risk factor indicates potential contributions from genetic or environmental influences. An extended duration of underlying myositis further amplifies the risk, increasing the necessity for early surveillance and intervention within vulnerable populations [32].

Pulmonary manifestations in PM/DM include a range of complications, especially ILD. Additional respiratory manifestations include ventilation defects due to weakness of the respiratory muscles and aspiration pneumonia resulting from involvement of the pharyngeal muscles [23]. ILD constitutes the predominant extra-muscular manifestation observed in myositis, with prevalence rates ranging from 19% to 40% among patients. The estimated incidence of ILD has been calculated at 1011 cases per 100,000 patient-years for those diagnosed with DM and 831 cases per 100,000 patient-years for individuals with PM [55]. Notably, pulmonary complications represent the principal cause of mortality within this group of systemic diseases [54]. Baseline assessment should include HRCT evaluation alongside pulmonary function tests for all of IIMs patients, especially for those with antisynthetase syndrome or anti-MDA5 antibodies [55]. ILD can often precede the clinical onset of myositis and is associated with increased mortality [1]. The clinical course of ILD in IIMs typically follows one of three patterns: an acute, rapidly progressive form; a chronic, slowly progressive form and an asymptomatic form. In a cohort of 107 patients with DM/PM-associated ILD, 18.7% presented with acute onset, 51.4% had progressive disease, and 29.9% were asymptomatic. Factors linked to ILD progression include older age at ILD onset, presence of dyspnea and cough at diagnosis, and acute symptomatic onset, lower baseline and follow-up lung function (FVC, DLCO) and a UIP pattern on HRCT [57].

**Figure 14:**
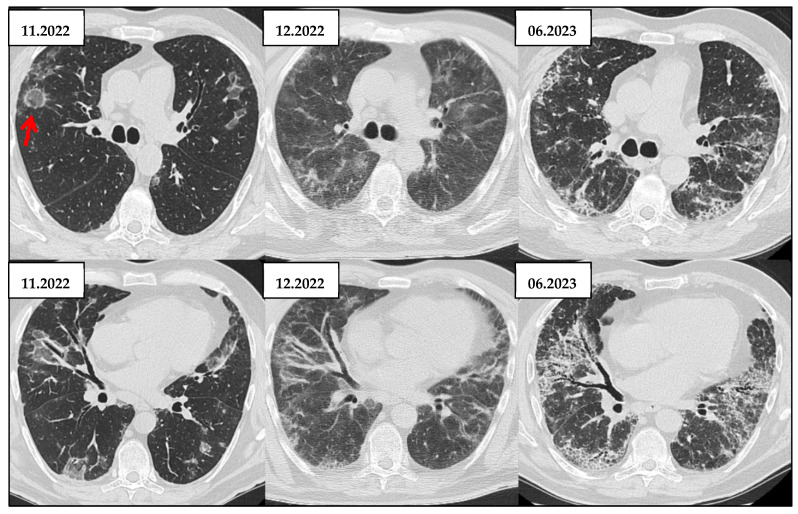
Radiological Evolution and Clinical Correlation in a Case of Dermatomyositis and Antisynthetase Syndrome-Associated ILD. A 67-year-old male underwent serial chest CT scans between November 2022 and June 2023, demonstrating progressive ILD later linked to DM with antisynthetase syndrome. In November 2022, the patient was diagnosed with SARS-CoV-2 infection. At that time, he exhibited no signs of myositis and was not further evaluated. HRCT from November 2022 revealed an OP pattern, characterized by multifocal consolidations and the presence of the “atoll sign” (reversed halo sign) (red arrow), consistent with post-viral or inflammatory changes. A follow-up scan in December 2022 (non-HRCT, 5 mm slices) showed partial resolution post-steroid therapy, though detailed interstitial evaluation was limited due to suboptimal image resolution. By June 2023, the patient developed progressive dyspnea, proximal muscle weakness and myalgia. He was subsequently diagnosed with dermatomyositis and antisynthetase syndrome, confirmed by positive anti-Jo-1 and anti-Ro-52 antibodies. HRCT at this stage revealed a NSIP pattern with bilateral, symmetric ground-glass opacities and reticulations, indicating a chronic fibrosing ILD process. These images demonstrate a radiological transition from an OP pattern to fibrotic NSIP over approximately seven months, correlating with the clinical onset of inflammatory myopathy, highlighting the importance of longitudinal imaging and consideration of autoimmune etiologies.

The most common HRCT pattern observed in PM/DM-associated ILD is NSIP. Over time, a significant proportion of patients develop fibrotic changes, with up to 67% showing fibrosis on follow-up imaging [53]. Less frequently, other HRCT patterns may be seen, such as OP and UIP. A combination of NSIP and OP features is also common (Figure 14). In our review, the distribution differed somewhat, with NSIP accounting for approximately 40% of cases, UIP observed in nearly 20%, and OP also representing about 20%. Rapidly progressive ILD typically presents with an acute interstitial pneumonia (AIP) pattern on HRCT and is associated with histopathological (HP) findings of diffuse alveolar damage (DAD), which carries a poor prognosis [55]. Patients with either DAD or UIP patterns have markedly reduced survival rates, with only about 33% surviving five years. In contrast, those with OP or NSIP patterns tend to have more favorable outcomes [1].

Table 7 provides a summary of features of IIMs-ILD discussed above.

#### 3.4.5. Mixed Connective Tissue Disease

Mixed Connective Tissue Disease (MCTD) is a pathology initially described by Sharp et al. in the year 1972, and is serologically distinguished by the detection of anti-U1 ribonucleoprotein (anti-U1 RNP) antibodies [41]. Clinically it manifests with overlapping characteristics of systemic lupus erythematosus, systemic sclerosis, and polymyositis. The most frequently clinical manifestations include Raynaud’s phenomenon, synovitis, inflammatory myopathy, and sclerodactyly. Therefore, the diagnosis of MCTD requires the presence of two or more of these clinical features in addition with positive anti-U1 RNP autoantibodies [52].

Pulmonary involvement is a significant manifestation, contributing substantially to both morbidity and mortality. The most common pulmonary complications include ILD and PAH, both of which have been recognized as major determinants of disease prognosis. The reported prevalence of ILD in MCTD varies considerably across studies, ranging from 27.4% to 78%, reflecting both the heterogeneity of study populations and diagnostic criteria [58]. ILD is now recognized as a principal factor in poor outcomes among MCTD patients. Predictors associated with ILD development include older age, the presence of Raynaud’s phenomenon, dysphagia, elevated CRP levels, anti-Ro52 autoantibodies, and capillaroscopic findings such as giant capillaries [59].

HRCT has identified NSIP as the predominant radiologic pattern, followed by UIP [32,59]. However, in our review, UIP was observed in nearly 50% of cases, NSIP in approximately 40%, and OP in about 5%, indicating some variation in pattern prevalence compared to previous reports. Still, the data are limited by the small number of patients diagnosed with MCTD included in this review. While ILD is relatively common in MCTD, it is often clinically mild in the majority of cases [60].

PAH, however, is a more severe manifestation and is considered the leading cause of mortality in MCTD. Although precise incidence rates remain uncertain due to a lack of large-scale studies, estimates suggest a prevalence ranging from 14% to 60% [60]. The variable clinical presentation and insidious onset of PAH necessitate a high index of suspicion and regular screening. Other thoracic manifestations include serositis, which is relatively common, with an estimated prevalence ranging from 6% to 50%. Pleural effusions, when present, are usually exudative in nature and may be transient or self-limited [60].

Table 8 provides a summary of features of MCTD-ILD discussed above.

### 3.5. Role of HRCT in Longitudinal Assessment of CTD-ILD

In addition to baseline analysis, HRCT fulfills an important role in the continuous monitoring of these patients, allowing assessment of disease progression. In accordance with current ACR/CHEST guidelines, the advised frequency of HRCT scanning varies depending on the specific CTD and individual clinical circumstances. For SSc, HRCT is recommended at baseline for all patients, with follow-up intervals guided by clinical evolution. In RA, MCTD, IIMs, and SS, HRCT at presentation is advised primarily for patients at higher risk, as previously described, with subsequent imaging performed as clinically indicated. Annual evaluations are suggested for higher-risk patients, while lower-risk individuals may be monitored less frequently, guided by PFTs and symptom progression [61]. While the guidelines do not explicitly tie HRCT frequency to WHO (The World Health Organization) functional class, patients exhibiting progressive respiratory symptoms or declining pulmonary function should be evaluated more frequently.

Repeat HRCT is particularly important for cases with ambiguous patterns, such as probable or indeterminate UIP, as serial assessments help clarify the diagnosis, improve prognostic insight, and guide treatment decisions.

These recommendations are supported by moderate-quality evidence (Level B) and are classified as Class IIa in the current ACR/CHEST guidelines [61].

According to the joint statement from the Portuguese Pulmonology, Rheumatology, and Radiology Societies, the frequency of HRCT monitoring in CTD-ILD should be specific for each disease. For SSc, HRCT is recommended at baseline and annually during the first 3–4 years, with more frequent imaging for patients showing severe or progressive pulmonary involvement. In RA, HRCT at presentation is advised for higher-risk patients, with follow-up scans typically performed annually or once every two years on clinical evolution. For IIMs, HRCT is recommended at baseline and then annually or biennially according to disease severity, while in SS, follow-up HRCT is usually performed every 2–3 years, or more frequently for high-risk patients. MCTD patients should have HRCT at baseline, with repeat imaging guided by phenotype and disease course, with SSc-like presentations warranting closer surveillance [62].

In patients with concomitant PAH, HRCT follow-up also plays a role by helping to discern whether clinical deterioration is driven by ILD progression or worsening pulmonary hypertension, a distinction with direct therapeutic implications.

## 4. Discussion

The findings of this review are consistent with previously published literature, demonstrating that the predominant HRCT patterns in CTD-ILDs align closely with those reported in other systematic reviews and meta-analyses. The prevalence of UIP and NSIP patterns observed in this study corroborates the established understanding that these represent the most common radiological manifestations across diverse CTDs. Our findings seem broadly consistent with the systematic review by Joy et al. (2023) [32], which reported NSIP as the predominant pattern across most CTD subtypes except for RA, where UIP was the most prevalent. This concordance reinforces the reliability of HRCT as a diagnostic tool and supports its continued use in clinical practice for the evaluation and management of CTD-ILDs. From a clinical perspective, recognition of these differential patterns is relevant in both diagnosis and prognosis, given the established association of UIP with poorer outcomes.

To illustrate the patterns described in the literature, we present representative cases from our clinical experience. These examples demonstrate how they manifest in patients with different CTDs and support the applicability of our review findings to real-world settings.

However, a notable limitation of this review is the relatively small number of cases exhibiting less common HRCT patterns such as LIP and OP, or patients with infrequent CTDs such as SS or MCTD. The constrained depiction may diminish the generalizability of the observations and highlights the necessity for more extensive, multicentric investigations to more accurately characterize HRCT patterns prevalence and clinical relevance. Furthermore, the heterogeneity of patient cohorts, disparities in imaging methodologies, and inconsistencies in pattern classification criteria among the studies examined may engender bias and influence the comparability of the findings. In particular, non-UIP cases that were not further specified were grouped under the category ‘Other,’ which may oversimplify heterogeneous radiological patterns. To improve consistency and data refinement, future studies should adopt updated guideline-based definitions of UIP and provide more descriptive categorization of non-UIP subtypes.

Future investigations should not only integrate imaging findings with relevant clinical parameters (such as disease duration, symptoms, and pulmonary function tests), serological markers, and histopathological information to develop predictive and prognostic models, but also rely on prospectively designed, multicenter cohorts with larger and more diverse populations to generate more generalizable and clinically meaningful conclusions.

Another limitation is that, given the cross-sectional or retrospective nature of the available studies, our review cannot establish causal relationships or determine the long-term prognostic value of different HRCT patterns in specific CTDs.

The inability to formally assess reporting bias represents a limitation of our review, as selective reporting or non-publication of results may have influenced the observed incidence of HRCT patterns across CTDs. Also, this review has some methodological limitations. The search was limited to three databases, and gray literature was not included. This systematic review was not registered in a prospective register; no protocol was prepared prior to the review and there were no amendments to report.

In summary, while this review confirms the predominance of UIP and NSIP patterns in CTD-ILDs as reported in the literature, the underrepresentation of less common patterns such as LIP and OP highlights an area for further investigation. Addressing these gaps will enhance our understanding of the full spectrum of HRCT manifestations in CTDs.

## 5. Conclusions

The aim of this review was not only to compile and analyze data from the existing literature regarding the frequency of various HRCT patterns across different CTD-ILDs, but also to summarize pertinent clinical and epidemiological information about the underlying CTDs themselves. By integrating radiological patterns with disease-specific characteristics, the review seeks to provide a comprehensive resource that is both informative for the reader and directly applicable to clinical practice.

While this review provides a comprehensive overview of HRCT patterns across CTD-ILDs, it is important to acknowledge that the findings are based primarily on retrospective and cross-sectional studies, and HRCT patterns were assessed in isolation. Future research should aim to develop predictive and prognostic models that integrate imaging findings with clinical parameters, serological markers, and histopathological information, ideally within prospective, multicenter cohorts encompassing larger and more diverse patient populations.

In conclusion, this review underscores the pivotal role of HRCT in the assessment of CTD-ILD, confirming that specific imaging patterns, such as UIP, NSIP, OP and LIP, are associated with distinct connective tissue diseases.

## Figures and Tables

**Table 1:** Inclusion/exclusion criteria.

Inclusion Criteria	Exclusion Criteria
Studies that were published in English between 2015 and 2024;	Studies written in languages other than English or published before 2015;
Trials involving adult participants (18 years and older);	Publications involving individuals under the age of 18;
Investigations conducted on human subjects;	Investigations conducted on non-human subjects;
Research specifically investigating CTD-ILDs;	Studies focusing on pathologies other than CTD-ILDs or including unrelated associated conditions;
Publications utilizing HRCT as the primary imaging method;	Research utilizing imaging methods other than HRCT.
Original studies with retrospective, prospective, or cross-sectional designs that provide extractable data.	Articles that are reviews, editorials, commentaries, case reports, or do not present original research data.

**Table 2:** Comparison of HRCT patterns among different CTD subtypes with Kruskal–Wallis and Dunn’s post hoc tests.

	IIM	MCTD	RA	SS	SSC	*p* ^#^
Definite UIP (%)	0.067 0.00090 to 0.26 n = 9 (a)	0.19 n = 4	0.48 0.36 to 0.53 n = 13 (a)	0.13 n = 5	0.25 0.11 to 0.42 n = 7	0.0004
Probable UIP (%)	0.00 0.00 to 0.058 n = 9	0.00 n = 4	0.00 0.00 to 0.075 n = 10	0.045 n = 4	0.00 0.00 to 0.059 n = 6	0.7547
Indeterminate UIP (%)	0.00 0.00 to 0.17 n = 9	0.00 n = 4	0.00 0.00 to 0.00 n = 10	0.045 n = 4	0.00 0.00 to 0.25 n = 6	0.7347
NSIP (%)	0.50 0.25 to 0.59 n = 9	0.63 n = 4	0.20 0.13 to 0.36 n = 10	0.29 n = 4	0.66 0.23 to 0.72 n = 6	0.0240
OP (%)	0.19 0.00 to 0.43 n = 9	0.00 n = 4	0.045 0.00 to 0.14 n = 10	0.14 n = 4	0.00 0.00 to 0.17 n = 6	0.2283
LIP (%)	0.00 0.00 to 0.00 n = 9 (a)	0.00 n = 4 (b)	0.00 0.00 to 0.0071 n = 10	0.064 n = 4 (a)(b)	0.00 0.00 to 0.081 n = 6	0.0158
Other (%)	0.17 0.00 to 0.37 n = 9	0.028 n = 4	0.18 0.079 to 0.52 n = 13	0.072 n = 5	0.00 0.00 to 0.42 n = 7	0.3303

Data are: Median, 95% Confidence Interval, n = number of studies included, Kruskal–Wallis test. (a)/(b)—groups with the same letter on the same row differ significantly in the post hoc test according to Dunn. # *p*-value refers to statistical significance for group comparison.

**Table 3:** Radiologic features of common ILD patterns.

	UIP	NSIP	LIP	OP
**Bilateral**	Yes	Yes	Yes	Yes
**Apicobasal gradient**	Yes	Yes	Yes	-
**Peripheral**	Yes	Yes *	-	Yes
**Peribronchovascular**	-	Yes	Yes	Yes
**Honeycombing**	Yes	Minimal	-	-
**Ground-glass**	Minimal	Yes	Yes	Yes
**Bronchiectasis**	Yes	Yes	-	-
**Nodules**	-	-	Yes	Yes
**Cysts**	-	-	Yes	-
**Consolidation**	-	-	-	Yes

*—with subpleural sparing.

**Table 4:** Key clinical, radiological, and prognostic features of systemic sclerosis–associated interstitial lung disease (SSc-ILD).

Category	Key Characteristics	References
**Epidemiology**	- Rare disease: ~10 cases/million/year - Female predominance (4:1) - Onset between ages 30–70	[1,26]
**ILD**	- Leading cause of death in SSc (≈ 1/3 of SSc-related mortality) - 64% by HRCT vs. 22% by chest X-ray, demonstrating HRCT’s superior sensitivity	[26,27]
**Risk Factors for ILD in SSc**	- Anti-SCL-70 and speckled ANA positivity - Anti-TRIM21 positivity - Male gender - African American race - Diffuse cutaneous form - Early disease (first 5–7 years) - Elevated acute phase reactants	[28]
**Clinical Presentation**	- Asymptomatic in early/mild stages - Later: exertional dyspnea, persistent dry cough, “velcro” crackles	[1,10]
**Physiological Findings**	- Restrictive ventilatory dysfunction - Decreased DLCO	[1]
**Diagnostic Recommendations**	- 2024 BSR guideline recommends baseline HRCT for all newly diagnosed SSc patients - PFTs alone may miss early-stage ILD - HRCT is essential for early detection	[29,30]
**HRCT Patterns**	- NSIP (~45–75%): ground-glass opacities, lower lobe predominance - UIP (~20–25%): honeycombing, traction bronchiectasis, worse prognosis - OP (~30%) - Other patterns (~5%)	[32]
**Pulmonary Arterial Hypertension**	- Prevalence up to 15%—SSc has the highest PAH risk among CTDs - Risk factors: advanced age, lcSSc subtype, disease duration >3 yrs, low DLCO (FVC/DLCO > 1.6), anti-centromere antibodies, elevated BNP/NT-proBNP, uric acid, telangiectasias - HRCT findings: pulmonary artery diameter ≥29 mm, PA/A ratio >1, right heart chambers dilation	[34,35]
**Esophageal Involvement**	- Affects ~90% of SSc patients - Early sign on HRCT: dilated esophageal lumen (1.2–4 cm, mean 2.3 cm) - Can lead to aspiration pneumonia due to dysmotility and reflux	[10,37]
**Lung Cancer Risk**	- Increased incidence in SSc (~10.7%) - SSc smokers have a 7-fold increased risk of lung cancer compared to non-SSc smokers	[37]

**Table 5:** Key clinical, radiological, and prognostic features of rheumatoid arthritis-associated interstitial lung disease (RA-ILD).

Category	Key Characteristics	References
**Epidemiology**	- 0.5 to 1% of the adult population - female-to-male ratio of about 2–3:1 - 25–50 years of age	[38]
**ILD**	- Clinically evident ILD occurs in ~10% of RA patients; up to 30% show subclinical ILD on HRCT - RA-ILD is more prevalent in men. - x3 increase in mortality	[8,40]
**Risk Factors for ILD in RA**	- Risk increases with age - Male sex - Smoking history (>25 pack-years) - High RA activity with RF and anti-CCP positivity - Longer disease duration	[42]
**Clinical Presentation**	- Exertional dyspnea, dry cough, and velcro crackles - In some cases, ILD may precede joint symptoms - Diagnosis may be incidental on HRCT.	[28,43]
**Diagnostic Recommendations**	- HRCT—not for everyone at baseline - Risk-based approaches	[42]
**HRCT Patterns**	- UIP is the most common pattern (~40–60%)—strong negative prognostic marker - NSIP (~15–25%) - OP and mixed types less frequent. (<20%)	[43]
**Distinctive HRCT Signs**	- Anterior upper lobe (AUL) sign - Straight-edge (SE) sign - Exuberant honeycombing (EHC)	[45]
**Pulmonary Nodules**	- Occur in ~20% of RA patients. 0.5–5 cm, peripherally within the mid and upper pulmonary zones - More commonly in men, smokers, and those with subcutaneous nodules or high RF titers - Cavitation and complications like pneumothorax may occur	[38]
**Small Airways Disease**	- Constrictive bronchiolitis (CB)—irreversible airflow limitation, seen on HRCT as mosaic attenuation, air trapping, bronchial wall thickening and centrilobular nodules - Follicular bronchiolitis (FB) presents with centrilobular nodules, tree-in-bud pattern and ground-glass opacities in a peribronchial distribution—responds to steroids	[38]

**Table 6:** Key clinical, radiological, and prognostic features of primary Sjögren syndrome-associated interstitial lung disease (pSS-ILD).

Category	Key Characteristics	References
**Epidemiology**	- 3.9–5.3 per 100,000 person-years. - strong female predominance (9–13:1). - mean diagnosis age of ~56.	[46]
**ILD**	- occurs in ~16% of pSS patients. - subclinical HRCT abnormalities are found in up to 65% of asymptomatic individuals.	[47]
**Risk factors for ILD in pSS**	- older age, male sex, smoking history, high ESSDAI score, long disease duration, elevated inflammatory markers (CRP, ESR), autoantibody positivity (Anti-Ro/SSA, especially Ro-52; Anti-La/SSB), and hypergammaglobulinemia.	[21,32,49]
**Clinical presentation**	- dyspnea, cough (with or without sputum), and chest pain. - symptom severity ranges from asymptomatic to severe respiratory compromise.	[47]
**Diagnostic recommendations**	- chest X-ray and PFT. - HRCT when pulmonary involvement is suspected.	[21,47]
**HRCT patterns**	- NSIP (42%) is most common, followed by UIP (11%), and OP/LIP (4% each). - some studies report higher LIP prevalence (up to 17.4%). - mixed patterns (e.g., NSIP + OP/LIP) are also frequently observed.	[21,50]
**Other pulmonary involvement**	- cystic lung disease (more common in pSS than other CTDs), - airway-centered abnormalities (centrilobular nodules, tree-in-bud), bronchiectasis, air trapping, mosaic attenuation—often linked to follicular or constrictive bronchiolitis.	[21,46,47,48]

**Table 7:** Key clinical, radiological, and prognostic features of idiopathic inflammatory myopathies–associated interstitial lung disease (IIMs-ILD).

Category	Key Characteristics	References
**Epidemiology**	- DM and PM are rare disorders—annual incidence of less than 10 cases per million individuals. - ILD—19–40% of cases (Incidence in DM: 1011/100,000 patient-years; in PM: 831/100,000 patient-years).	[52,55]
**Classification**	- IIMs include PM, DM, sIBM, nonspecific myositis, IMNM and ADM, Antisynthetase syndrome (ASyS)	[51,53]
**Clinical Presentation**	- proximal muscle weakness, elevated serum levels of muscle enzymes, myopathy, inflammatory infiltrates within muscle tissue, myositis-specific autoantibodies. - DM: Gottron’s papules, heliotrope rash and mechanic’s hands - ASyS: ILD, myositis, non-erosive seronegative arthritis, Raynaud’s phenomenon, mechanic’s hands, persistent fever and anti-ARS antibodies	[52,53,54]
**Risk Factors for ILD in IIMs**	- older age, Black race, elevated ESR/CRP, longer disease duration, presence of dyspnea/cough at diagnosis. - anti-MDA5 and anti-PL7/PL12 linked with severe or RP-ILD.	[32,56]
**Diagnostic Recommendations**	- all IIMs patients should undergo HRCT and pulmonary function tests at baseline and autoantibody testing.	[55]
**HRCT Patterns**	- NSIP 40%, UIP 20%, and OP 20% (according to this review). - combination of NSIP/OP can be seen. - AIP with HP findings of DAD is seen in acute RP-ILD.	[53,55]

**Table 8:** Key clinical, radiological, and prognostic features of mixed connective tissue disease-associated interstitial lung disease (MCTD-ILD).

Category	Key Characteristics	References
**Diagnosis**	- positive anti-U1 RNP autoantibodies; - at least two clinical features: Raynaud’s phenomenon, synovitis, inflammatory myopathy or sclerodactyly	[52]
**Pulmonary Involvement**	- ILD and PAH; - ILD prevalence ranges from 27.4% to 78%; - PAH prevalence ranges from 14 to 60%.	[58,60]
**ILD Risk Factors**	- older age, Raynaud’s phenomenon, dysphagia, elevated CRP, anti-Ro52 autoantibodies, and capillaroscopic abnormalities such as giant capillaries	[59]
**HRCT Patterns**	- predominantly NSIP, followed by UIP - in our review, UIP was observed in nearly 50% of cases, NSIP in approximately 40%, and OP in about 5%	[32,59]
**Other Thoracic Manifestations**	- serositis occurs in 6–50%; - pleural effusions typically exudative, transient, and self-limited	[60]

## Data Availability

The data supporting the findings of this review are included in the article; further details may be provided by the first or corresponding author upon request.

## Supplementary Materials

The following supporting information can be downloaded at: https://www.mdpi.com/article/10.3390/jcm14176164/s1, Table S1: Newcastle-Ottawa Assessment Scale for cohort studies; Table S2: Newcastle-Ottawa Assessment Scale for case-control studies; Table S3: Risk of Bias Assessment of Cross-Sectional Studies Using the JBI Checklist.

## Abbreviations

The following abbreviations are used in this manuscript:

A	Aorta
ACPAs	Anti-citrullinated peptide antibodies
ADM	Amyopathic dermatomyositis
AIP	Acute interstitial pneumonia
ALAT	Latin American Thoracic Association
Anti-MDA5	Anti-melanoma differentiation-associated gene 5
ARS	Aminoacyl-transfer RNA synthetases
ASyS	Antisynthetase syndrome
ATS	American Thoracic Society
AUL	Anterior Upper Lobe
BALT	Bronchus-associated lymphoid tissue
BNP	Brain natriuretic peptide
BSR	British Society for Rheumatology,
CB	Constrictive bronchiolitis
COP	Cryptogenic organizing pneumonia
CRP	C-reactive protein
CTD-ILDs	Connective tissue disease associated interstitial lung disease
CTDs	Connective tissue diseases
DAD	Diffuse alveolar damage
DLCO	Diffusion capacity for carbon monoxide
DM	Dermatomyositis
EHC	Exuberant Honeycombing
ERS	European Respiratory Society
ESR	Erythrocyte sedimentation rate
ESSDAI	EULAR Sjögren’s syndrome disease activity index
FB	Follicular bronchiolitis
FVC	Forced vital capacity
GGO	Ground-glass opacities
HP	Histopathological
HRCT	High-resolution computed tomography
IIMs	Idiopathic Inflammatory Myopathies
ILD	Interstitial lung disease
IMNM	Immune-mediated necrotizing myopathy
iNSIP	Idiopathic NSIP
IPF	Idiopathic pulmonary fibrosis
JRS	The Japanese Respiratory Society
lcSSc	Limited cutaneous SSc
LIP	Lymphoid interstitial pneumonia
MALT	Mucosa-associated lymphoid tissue
MCTD	Mixed Connective Tissue Disease
mMRC	Modified Medical Research Council
mPAP	Mean pulmonary arterial pressure
MSAs	Myositis-specific autoantibodies
NOS	Newcastle–Ottawa Scale
NSIP	Non-specific interstitial pneumonia
NT-proBNP	N-terminal pro brain natriuretic peptide
OP	Organizing pneumonia
PA	Pulmonary artery
PAH	Pulmonary arterial hypertension
PAWP	Pulmonary arterial wedge pressure
PFT	Pulmonary function tests
PM	Polymyositis
PRISMA	Preferred Reporting Items for Systematic Reviews and Meta-Analyses
pSS	Primary Sjögren’s syndrome
PVR	Pulmonary vascular resistance
RA	Rheumatoid Arthritis
RF	Rheumatoid factor
RHC	Right heart catheterization
RNP	Ribonucleoprotein
RP-ILD	Rapidly progressive interstitial lung disease
SE	Straight-Edge
sIBM	Sporadic inclusion-body myositis
SLE	Systemic Lupus Erythematosus
SS	Sjögren’s syndrome
SSc	Systemic Sclerosis
TGF-β	Transforming growth factor-beta
UIP	Usual interstitial pneumonia
WHO	The World Health Organization

## Appendix A

**Table A1:** Main characteristics of included studies.

Type of CTD	Study	Design	Country	n	HRCT–ILD, n (%)	Definite UIP, n (%)	Probable UIP, n (%)	Indeterminate UIP, n (%)	NSIP, n (%)	OP, n (%)	LIP, n (%)	Other, n (%)
**Systemic Sclerosis**	Agarwal et al., 2021, [63]	Cross-sectional	India	21	21 (100%)	3 (14.29%)	0	0	14 (66.67%)	4 (19.05%)	0	0
	Ibrahim et al., 2020, [64]	Cross-sectional	Egypt	30	20 (66.6%)	5 (25%)	0	0	13 (65%)	0	2 (10%)	0
	Kaur et al., 2024, [17]	Cross-sectional	India	3	3 (100%)	1 (33.3%)	0	0	2 (66.6%)	0	0	0
	Nurmi et al., 2023, [65]	Retrospective	Finland	15	15 (100%)	3 (20%)	1 (6.7%)	3 (20%)	2 (13.3%)	1 (6.7%)	0	5 (33.3%)
	Oliveira et al., 2022, [2]	Retrospective	Portugal	26	26 (100%)	7 (27%)	0	0	19 (73%)	0	0	0
	Yamakawa et al., 2020, [66]	Retrospective	Japan	38	38 (100%)	3 (7.89%)	1 (2.65%)	10 (26.31%)	24 (63.15%)	0	0	0
	Yıldırım et al., 2019, [67]	Retrospective	Turkey	18	18 (100%)	9 (50%)						Non-UIP = 9 (50%)
**Rheumatoid arthritis**	Agarwal et al., 2021, [63]	Cross-sectional	India	26	26 (100%)	10 (38.47%)	0	0	8 (30.77%)	4 (15.38%)	0	4 (15.38%)
	Kaur et al., 2024, [17]	Cross-sectional	India	4	4 (100%)	2 (50%)	0	0	2 (50%)	0	0	0
	Kim et al., 2020, [68]	Retrospective	Korea	153	153 (100%)	59 (38.6%)						Non UIP = 94 (61.4%)
	Lee et al., 2021, [40]	Retrospective/prospective	Korea	60	60 (100%)	31 (51.67%)	0	0	18 (30%)	0	0	11 (18.33%)
	Nurmi et al., 2016, [43]	Retrospective	Finland	59	59 (100%)	35 (59.34%)	0	0	8 (13.55%)	7 (11.86)	0	9 (15.25%)
	Nurmi et al., 2023, [65]	Retrospective	Finland	71	71 (100%)	37 (52.1%)	5 (7%)	0	7 (9.9%)	9 (12.7%)	0	13 (18.3%)
	Oh et al., 2022, [69]	Retrospective	Korea	158	144 (91.13%)	53 (36.8%)						Non UIP = 91 (63.2%)
	Oliveira et al., 2022, [2]	Retrospective	Portugal	15	15 (100%)	8 (53.34%)	0	0	6 (40%)	0	0	1 (6.66%)
	Ren et al., 2023, [39]	Retrospective	China	154	67 (43.5%)	41 (61.2%)	0	0	10 (14.9%)	2 (3%)	0	14 (20.9%)
	Yamakawa et al., 2019, [70]	Retrospective	Japan	96	96 (100%)	20 (21%)	19 (20%)	29 (30%)	13 (14%)	6 (6%)	0	9 (9%)
	Yang et al., 2019, [71]	Retrospective	Korea	77	67 (87%)	32 (47.7%)	0	0	17 (25.4%)	13 (19.4%)	2 (3%)	3 (4.5%)
	Yıldırım et al., 2019, [67]	Retrospective	Turkey	28	28 (100%)	8 (28.6%)						Non UIP = 20 (71.4%)
	Yunt et al., 2017, [72]	Retrospective	USA	385	292 (75.84%)	100 (34.28%)	23 (7.87%)	0	35 (11.98%)	4 (1.36%)	4 (1.36%)	126 (43.15%)
**Sjögren’s Syndrome**	Agarwal et al., 2021, [63]	Cross-sectional	India	16	16 (100%)	2 (12.50%)	0	0	7 (43.75%)	5 (31.25)	1 (6.25%)	1 (6.25%)
	Gao et al., 2018, [50]	Retrospective	China	165	69 (41.81%)	11 (15.95%)	0	13 (18.8%)	27 (39.13%)	1 (1.44%)	12 (17.4%)	5 (7.24%)
	Kim et al., 2021, [5]	Retrospective	Korea	62	62 (100%)	24 (38.75%)	26 (41.9%)	0	7 (11.29%)	1 (1.61%)	4 (6.45%)	0
	Nurmi et al., 2023, [65]	Retrospective	Finland	11	11 (100%)	1 (9.1%)	1 (9.09%)	1 (9.09%)	2 (18.18%)	3 (27.27)	0	3 (27.27%)
	Yldırım et al., 2019, [67]	Retrospective	Turkey	9	9 (100%)	1 (11.1%)						Non UIP = 8 (88.9%)
**Idiopathic Inflammatory Myopathies**	Abel et al., 2023, [53]	Retrospective	France	72	58 (80.55%)	4 (6.9%)	0	0	29 (50%)	3 (5.17%)	0	22 (37.93%)
	Agarwal et al., 2021, [63]	Cross-sectional	India	4	4 (100%)	0	0	0	1 (25%)	0	0	3 (75%)
	Chen et al., 2019, [73]	Retrospective	China	20	20 (100%)	0	0	0	11 (55%)	9 (45%)	0	0
	Chen et al., 2023, [56]	Retrospective	China/Japan	308	308 (100%)	2 (0.65%)	0	0	87 (28.25%)	142 (46.1%)	0	77 (25%)
	Nurmi et al., 2023, [65]	Retrospective	Finland	15	15 (100%)	1 (6.7%)	1 (6.7%)	1 (6.7%)	2 (13.3%)	5 (33.3%)	0	5 (33.3%)
	Oliveira et al., 2022, [2]	Retrospective	Portugal	5	5 (100%)	2 (40%)	0	0	3 (60%)	0	0	0
	Vojinovic et al., 2021, [74]	Retrospective	Italy	165	47 (28.48%)	6 (12.84%)	0	9 (19.14%)	15 (31.92%)	9 (19.1%)	0	8 (17%)
	Yamakawa et al., 2020, [66]	Retrospective	Japan	24	24 (100%)	1 (4%)	2 (8%)	5 (21%)	16 (67%)	0	0	0
	Zanatta et al., 2023, [75]	Retrospective	Italy/France	253	78 (30.83%)	22 (28%)	0	0	39 (50%)	17 (22%)	0	0
**Mixed Connective Tissue Disease**	Agarwal et al., 2021, [63]	Cross-sectional	India	18	18 (100%)	3 (16.67%)	0	0	10 (55.56%)	4 (22.22)	0	1 (5.55%)
	Nurmi et al., 2023, [65]	Retrospective	Finland	7	7 (100%)	1 (14.28%)	1 (14.28%)	1 (14.28%)	1 (14.28%)	0	0	3 (42.88%)
	Oliveira et al., 2022, [2]	Retrospective	Portugal	7	7 (100%)	2 (29%)	0	0	5 (71%)	0	0	0
	Shan and Ge, 2024, [58]	Retrospective	China	59	39 (66.1%)	8 (20.5%)	0	0	31 (79.5%)	0	0	0
