# Supplementary material for: High-Resolution CT Findings in Interstitial Lung Disease Associated with Connective Tissue Diseases: Differentiating Patterns for Clinical Practice—A Systematic Review with Meta-Analysis
Source: J Clin Med. 2025 Aug 31;14(17):6164. doi: 10.3390/jcm14176164 (PMC12429569; doi:10.3390/jcm14176164)
Supplement: Supplementary file 1 [file jcm-14-06164-s001.zip › jcm-3831158-supplementary.pdf]

Table S1. Newcastle-Ottawa Assessment Scale for cohort studies.

Study	Selection (4)				Comparability (2)		Outcome (3)			Total score	Risk of Bias
Abel et al., 2023	★	☆	★	★	★	☆	★	★	★	7	Low
Chen et al., 2019	★	☆	★	★	★	☆	★	★	★	7	Low
Chen et al., 2023	★	★	★	★	★	★	★	★	☆	8	Low
Kim et al., 2020	★	☆	★	★	★	☆	★	★	★	7	Low
Kim et al., 2021	★	★	★	★	★	☆	★	★	★	8	Low
Lee et al., 2021	★	☆	★	★	☆	☆	★	★	★	6	Moderate
Nurmi et al., 2016	★	★	★	★	☆	☆	★	★	★	7	Low
Nurmi et al., 2023	★	★	★	★	★	☆	★	☆	★	7	Low
Oh et al., 2022	★	☆	★	★	★	☆	★	★	★	7	Low
Oliveira et al., 2022	★	★	★	★	★	☆	★	★	★	8	Low
Shan and Ge, 2024	★	★	☆	★	☆	☆	★	★	☆	5	Moderate
Vojinovic et al., 2021	★	★	☆	★	☆	☆	★	★	☆	5	Moderate
Yamakawa et al., 2019	★	★	☆	★	★	☆	★	★	☆	6	Moderate
Yamakawa et al., 2020	★	★	☆	★	★	☆	★	★	☆	6	Moderate
Yang et al., 2019	★	★	★	★	★	★	★	★	★	9	Low
Yıldırım et al., 2019	★	★	★	★	★	☆	★	★	☆	7	Low
Yunt et al., 2017	★	★	★	★	★	★	★	★	★	9	Low
Zanatta et al., 2023	★	★	★	★	★	★	★	★	☆	8	Low

Table S2. Newcastle-Ottawa Assessment Scale for case-control studies.

Study	Selection (4)				Comparability (2)		Outcome (3)			Total score	Risk of Bias
Gao et al., 2018	★	★	★	★	★	☆	★	☆	☆	6	Moderate
Ren et al., 2023	★	★	★	★	★	☆	★	☆	☆	6	Moderate

Table S3. Risk of Bias Assessment of Cross-Sectional Studies Using the JBI Checklist

**Study**	**Q1: Target population clearly defined**	**Q2: Inclusion/ exclusion criteria clear**	**Q3: Sample representative**	**Q4: Exposure measured validly**	**Q5: Outcome measured validly**	**Q6: Confounders identified and controlled**	**Q7: Statistical analysis appropriate**	**Q8: Sufficient data reported**	**Overall risk of bias**
Agarwal et al., 2021	Yes	Yes	Yes	Yes	Yes	No	Yes	Yes	Low
Ibrahim et al., 2020	Yes	Yes	Yes	Yes	Yes	No	Yes	Yes	Low
Kaur et al., 2024	Yes	Yes	Yes	Yes	Yes	No	Yes	Yes	Low
